# Functional Status, Quality of Life and Survival After Repeated Intrathecal Administration of Autologous Lineage-Negative Cells in Amyotrophic Lateral Sclerosis

**DOI:** 10.3390/jcm15207937

**Published:** 2026-10-14

**Authors:** Monika Gołąb-Janowska, Agnieszka Wełnicka-Wesołowska, Bartłomiej Baumert, Karolina Machowska-Sempruch, Agnieszka Meller, Anna Sobuś, Edyta Paczkowska, Dariusz Kotlęga, Przemysław Nowacki, Bogusław Machaliński

**Affiliations:** 1 Department of Neurology, Pomeranian Medical University, 71-252 Szczecin, Poland; monika.golab.janowska@pum.edu.pl (M.G.-J.); agnieszka.welnicka.wesolowska@pum.edu.pl (A.W.-W.); karolina.machowska.sempruch@pum.edu.pl (K.M.-S.); agnieszka.meller@pum.edu.pl (A.M.); przemyslaw.nowacki@pum.edu.pl (P.N.); 2 Department of Hematology and Transplantology, Pomeranian Medical University, 71-252 Szczecin, Poland; 3 Department of General Pathology, Pomeranian Medical University, 70-111 Szczecin, Poland; anna.sobus@pum.edu.pl (A.S.); edyta.paczkowska@pum.edu.pl (E.P.); 4 University Hospitals of Northamptonshire, Northampton General Hospital NHS Trust, Northampton NN1 5BD, UK; 5 Department of Pharmacology and Toxicology, University of Zielona Góra, 65-417 Zielona Góra, Poland

**Keywords:** amyotrophic lateral sclerosis, lineage-negative cells, cell therapy, quality of life, ALSAQ-40, ALSFRS-R, Norris scale, survival, BDNF

## Abstract

**Background/Objectives:** Adjuvant cell-based strategies are still being explored in amyotrophic lateral sclerosis (ALS). **Materials and Methods:** In this open-label, single-arm study of intrathecal autologous bone marrow-derived lineage-negative (LIN^−^) cells, we report functional status, quality of life, and survival after three administrations at six-week intervals. A total of 45 patients with sporadic ALS were qualified: 42 received all three administrations and 23 completed the 24-week assessment schedule, while 19 stopped attending because of deterioration. **Results**: In the 23 completers, Norris scale scores were stable until week 13 and then declined (*p* = 0.00004), and the Amyotrophic Lateral Sclerosis Assessment Questionnaire 40 (ALSAQ-40) followed the same pattern (*p* = 0.0019), whereas the ALS Functional Rating Scale—Revised (ALSFRS-R) declined at a constant 0.5 points per month. In all 42 treated patients the Norris score fell during treatment (0.95 points per month; 95% CI 0.52–1.38), so the plateau was confined to completers, who had milder baseline disease. Dose-related differences in quality of life were confounded by baseline imbalance; survival analyses were exploratory. Cell number did not correlate with cerebrospinal fluid brain-derived neurotrophic factor (BDNF) after the third administration in the 39 patients with paired samples (ρ = 0.14). No adverse event was recorded in 126 procedures, but harms were not graded systematically. **Conclusions:** These exploratory findings do not demonstrate efficacy; a sham-controlled trial with blinded raters is required.

## 1. Introduction

Amyotrophic lateral sclerosis (ALS) is a progressive neurodegenerative disorder characterised by selective loss of upper and lower motor neurons in the motor cortex, brainstem and spinal cord [1,2]. Progressive muscle atrophy, weakness and spasticity are followed by bulbar and respiratory involvement, and respiratory failure remains the most frequent cause of death [1,3]. Median survival from diagnosis is 3–5 years, although the phenotype and the rate of progression are strikingly heterogeneous [4,5,6].

The pathogenesis of ALS is multifactorial. Oxidative stress, glutamate excitotoxicity, mitochondrial dysfunction, disturbed RNA metabolism and protein aggregation act in concert with a maladaptive glial and immune response within the central nervous system (CNS) [7,8,9,10,11]. Despite three decades of translational effort, the pharmacological armamentarium remains modest: riluzole prolongs survival by a few months [12], edaravone slows functional decline in a narrowly defined subgroup [13], tofersen is restricted to the fewer than 2% of patients carrying *SOD1* mutations [14], and sodium phenylbutyrate–taurursodiol was withdrawn from the market in 2024 after a confirmatory phase III trial failed to reproduce the original signal [15]. The gap between what is understood about ALS biology and what can be offered to patients therefore remains wide [8].

Cell-based therapies address this gap from a different direction. Rather than targeting a single molecular pathway, transplanted bone marrow-derived cells are thought to act through paracrine mechanisms—secretion of neurotrophic factors that support motor neuron survival, and modulation of the local inflammatory milieu [16,17,18,19]. Autologous bone marrow mononuclear cells (MNCs) and their subpopulations are attractive because they are accessible, non-immunogenic and free of the ethical and oncological concerns attached to pluripotent sources [17,20,21]. Intrathecal delivery of bone marrow-derived cells has been shown to be feasible and safe in a series of phase I and II studies [20,22,23,24,25,26], and mesenchymal stromal cell products engineered to secrete neurotrophic factors have reached phase III [27,28,29]. The consistent message from that literature is instructive: primary endpoints have not been met in unselected populations, yet prespecified subgroups with earlier or slower-progressing disease have repeatedly shown signals of benefit, and repeated rather than single dosing appears to matter [25,27,28,29,30].

Within this framework our group has focused on the lineage-negative (LIN^−^) fraction of autologous bone marrow—an early, immunomagnetically enriched stem/progenitor population depleted of committed haematopoietic lineages, with documented capacity to secrete neurotrophins and to modify complement and neutrophil-dependent inflammatory pathways [31,32,33,34,35,36,37]. In previous reports from the same clinical programme, we described the safety and feasibility of single-LIN^−^ cell administration together with the associated humoral and microRNA profiles [31], the local and systemic humoral response [32], the immunological consequences of repeated administration [33], the silencing effect on the complement-mediated immune system [34], and the effect on articulatory and phonatory function [35,36,37]. What has not yet been reported is the clinical dimension of the same experiment over an extended horizon. No clinical, functional or quality-of-life outcome beyond day 28 has been reported from this cohort in any of those papers, and health-related quality of life has not been reported at all. The present analysis therefore addresses how functional status and above all patients’ own perception of their health evolved during and after a course of three intrathecal administrations, and whether the amount of bone marrow material obtained and administered had any bearing on those outcomes.

Two questions motivated the present analysis. First, is there a temporal relationship between the treatment period and the trajectory of validated clinical and quality-of-life instruments over 24 weeks of follow-up? Second, given the absence of a control group, does the biological ‘exposure’—the total yield of isolated MNCs and the total number of administered LIN^−^ cells—behave as an internal gradient that discriminates outcome, or does a plateau effect exist above which additional cells confer no advantage? To answer these questions we analysed Norris scale, ALSFRS-R and ALSAQ-40 trajectories in patients who completed a 24-week assessment schedule, related them to post hoc strata defined by cell yield and cell dose, and followed all treated patients for survival up to week 90. Because completion of the schedule depended on clinical status, we additionally analysed all treated patients with available data up to week 16 as a sensitivity analysis.

## 2. Materials and Methods

### 2.1. Study Design and Ethics

The study was a prospective, open-label, non-randomised, single-centre clinical trial in patients with probable or definite sporadic ALS diagnosed according to the revised El Escorial criteria [38]. The trial was registered at ClinicalTrials.gov (NCT02193893) and approved by the Local Bioethics Committee of the Pomeranian Medical University in Szczecin (approval code KB-0012/06/10, 25 January 2010). Patients were recruited and treated between February 2018 and February 2019, and were followed up for two years. All participants gave written informed consent before each procedure. The study was conducted in accordance with the Declaration of Helsinki. The report follows the Transparent Reporting of Evaluations with Nonrandomized Designs (TREND) statement for non-randomised evaluations; the completed TREND checklist is provided in the Supplementary Materials (Table S1).

Given the exploratory character of the programme and the absence of any established cellular therapy standard at the time of design, a placebo-controlled arm was not included. All participants continued riluzole and standard supportive care throughout. Autologous LIN^−^ cells were administered on three occasions at six-week intervals, each administration being followed by a seven-day hospitalisation. Patients were assessed 28 days after each administration and subsequently at three-month intervals.

**Figure 1:**
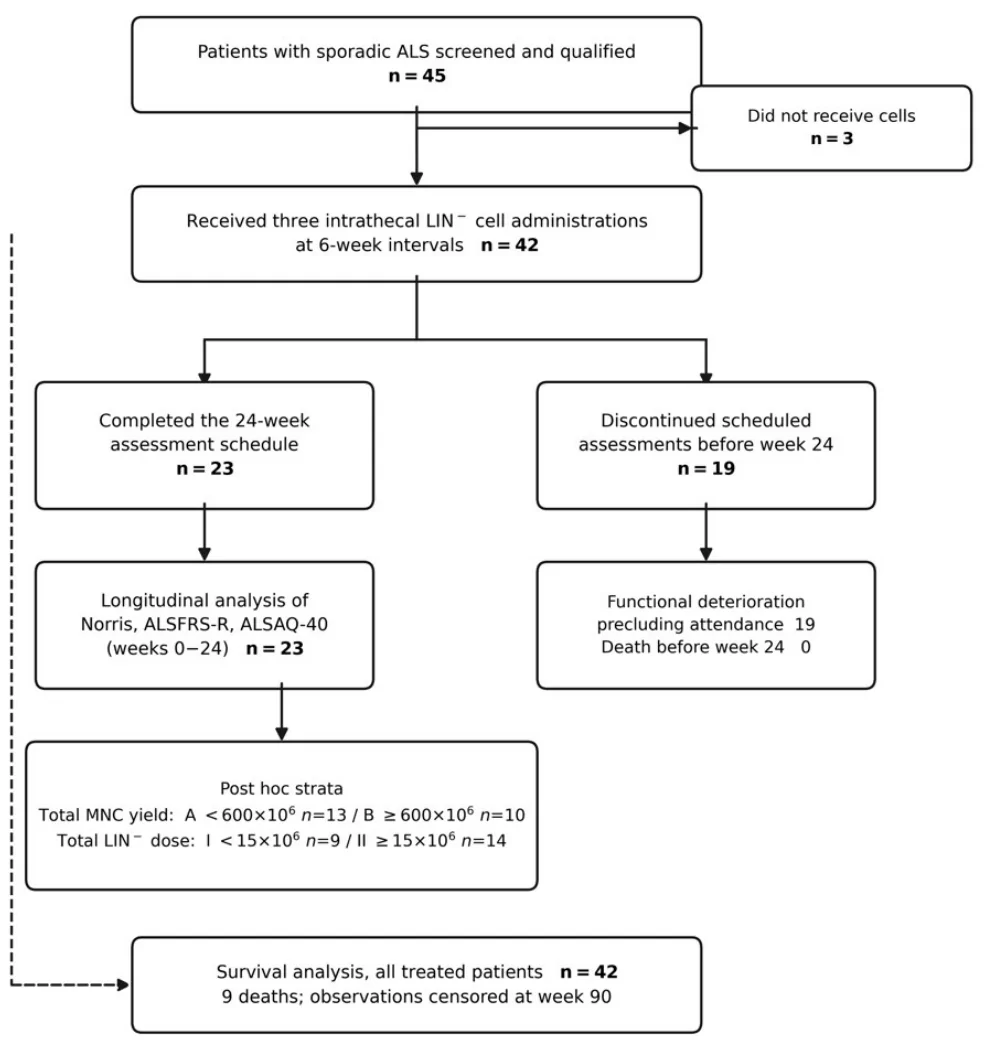
Flow of participants through the study. Forty-five patients with sporadic ALS were qualified; 42 received all three intrathecal LIN^−^ cell administrations. Twenty-three patients completed the predefined 24-week assessment schedule and formed the longitudinal efficacy cohort used for all analyses of clinical scales and quality of life; the remaining 19 discontinued scheduled assessments because functional deterioration precluded attendance, and no deaths occurred before week 24. All 42 treated patients were included in the survival analysis, with observations censored at week 90. LIN^−^—lineage-negative; MNC—mononuclear cell.

Two distinct analysis populations were defined for the present report and are shown in Figure 1. The longitudinal efficacy cohort comprised patients who completed the full 24-week assessment schedule and therefore contributed data at every predefined time point. This cohort was used for all analyses of clinical scales and quality of life. The survival population comprised all patients who received all three administrations, with the endpoint defined as death from any cause and observations censored at week 90. A third population, used for post hoc sensitivity analyses (Section 3.8), comprised all 42 treated patients irrespective of completion of the schedule.

### 2.2. Participants

Forty-five patients with sporadic ALS were qualified by the study board. Inclusion criteria were: age 18–65 years; capacity to give informed consent; probable or definite sporadic ALS according to the revised El Escorial criteria; no family history of ALS; at least three months of observation and riluzole treatment before the first cell administration; forced vital capacity >50% of predicted; and absence of significant comorbidity, including malignancy, diabetes mellitus, cardiovascular disease, chronic kidney or liver disease, or the use of drugs with known myelotoxic potential.

Patients were not stratified a priori according to cell dose or MNC yield. All subgroup comparisons reported here are therefore post hoc, exploratory and hypothesis-generating.

Sporadic ALS was defined clinically: patients had no family history of ALS and met the revised El Escorial criteria for probable or definite ALS, which by definition require exclusion of other disease processes that could explain the clinical and electrophysiological findings [38]. Diagnosis and eligibility were confirmed by the study board. Genetic testing for ALS-associated variants (for example, SOD1, C9orf72, TARDBP and FUS) was not part of the study protocol and was not performed systematically. The classification as sporadic therefore rests on family history and clinical criteria alone, and a genetic form cannot be excluded, particularly in the youngest participant (27 years) [39]. As part of the qualification work-up, all patients underwent electromyography with nerve conduction studies and magnetic resonance imaging to exclude alternative diagnoses. Beyond these investigations, a standardised laboratory panel for the exclusion of ALS mimics was not prescribed by the protocol. Upper and lower motor neuron involvement was not quantified with a dedicated instrument; the only phenotypic descriptors available are the site of onset (limb or bulbar) and the functional scales. Cognitive and behavioural function was not assessed formally at baseline or during follow-up. Capacity to give informed consent was required for inclusion, which excludes advanced cognitive impairment, but not milder ALS-related cognitive or behavioural change [40].

### 2.3. Clinical Assessment

Baseline data comprised age, sex, disease duration, site of onset, comorbidities and concomitant treatment. Functional status was quantified with two complementary instruments: the Norris scale [41], a 21-item examination-based scale offering fine-grained resolution of motor deficit, and the revised ALS Functional Rating Scale (ALSFRS-R) [42], the 48-point instrument accepted as the standard efficacy measure in ALS trials, for which a validated Polish version is available [43]. The Norris scale is no longer in routine use in registration trials, and we retained it for two reasons: it is administered by physical examination rather than by interview and resolves smaller increments of motor deficit than the ALSFRS-R, and it provides continuity with the earlier reports from this programme [31,32,33]. The two instruments are therefore not interchangeable, and the consequences of that difference are addressed in the Discussion. The Norris scale was assessed immediately before each treatment cycle and on days 1, 2, 3, 5, 7, 12, 18 and 28 after each administration, and thereafter at every follow-up visit. The ALSFRS-R was assessed at baseline, at discharge and at every follow-up visit.

Health-related quality of life was assessed with the ALS Assessment Questionnaire (ALSAQ-40) [44], a disease-specific patient-reported instrument comprising five domains—physical mobility, activities of daily living and independence, eating and drinking, communication, and emotional reactions. Domain and summary scores are transformed to a 0–100 scale on which higher values denote worse quality of life. The ALSAQ-40 was completed at baseline, at discharge and at every follow-up visit.

For the present report, results are anchored to the following time points relative to the first administration: baseline (week 0), weeks 1, 4, 6, 7, 10, 12, 13, 16 and 24. The second administration took place at the beginning of week 6 and the third at the beginning of week 12. Week 13 therefore represents the last on-treatment assessment, and weeks 16 and 24 the post-treatment period.

### 2.4. Bone Marrow Harvest, LIN^−^ Cell Isolation and Intrathecal Administration

Bone marrow harvest, LIN^−^ cell isolation and intrathecal administration were performed exactly as described previously [31,33]. In brief, bone marrow was aspirated from the posterior iliac crest under local anaesthesia. The total volume of aspirate with anticoagulant ranged from 44 to 156 mL (mean 122 ± 22.7 mL). MNCs were isolated by density gradient centrifugation in lymphocyte separation medium (MP Biomedicals, Santa Ana, CA, USA). LIN^−^ cells were then obtained by immunomagnetic negative selection using a commercial lineage cell depletion kit (Miltenyi Biotec, Auburn, AL, USA) according to the manufacturer’s instructions, and suspended in 2 mL of sterile phosphate-buffered saline (Polfa, Kutno, Poland). Intrathecal administration was performed by lumbar puncture at the L3/L4 or L4/L5 interspace, followed by post-procedural monitoring according to institutional protocol.

It should be emphasised that MNCs themselves were not administered to patients. The MNC count represents the yield obtainable from a given patient’s marrow and served in the present analysis as a surrogate of marrow cellularity and indirectly of host biological reserve.

#### Product Characterisation

Isolation was performed in a closed isolator system (Xvivo, BioSpherix, Parish, NY, USA) under good-manufacturing-practice conditions, as described previously [33]. Identity and purity of the LIN^−^ population were defined by depletion of cells expressing committed haematopoietic lineage antigens with the commercial lineage-depletion kit. The immunophenotype of LIN^−^ populations characterised in our earlier work (CD34+ 12.1 ± 7.2%, CD133+ 12.3 ± 8.2%, CD34+ CD133+ CD144+ endothelial progenitor cells 1.7 ± 1.1%) has been reported [19], and the flow-cytometry method applied to the ALS product (staining for CD34, CD133 and CD144, 10,000 events acquired) has been described [35]. When the same isolation procedure was used to prepare autologous LIN^−^ cells for intracoronary infusion, viability by trypan blue exclusion exceeded 95% [45]. Numerical release criteria and per-procedure viability, sterility and endotoxin results are not available in the trial database analysed here, and no potency assay (for example secretome profiling or colony-forming capacity) was performed. The immunophenotype cited above was obtained in earlier cohorts and is not a release test of the products administered in the present study.

### 2.5. Cerebrospinal Fluid Analysis

Cerebrospinal fluid (CSF) was collected immediately before each administration (day 0) and on day 7 of each treatment cycle. Concentrations of brain-derived neurotrophic factor (BDNF), nerve growth factor beta (NGF-β), neurotrophin 3 (NT-3), tumour necrosis factor alpha (TNF-α), TNF receptor (TNF-R) and C-reactive protein (CRP) were measured by multiplex fluorescent bead-based immunoassay (Luminex Corporation, Austin, TX, USA) according to the manufacturer’s protocol, as detailed in our earlier reports [31,32,33]. Because the humoral, immunological and microRNA data from this programme have been published in full elsewhere [31,32,33,34], the present report is confined to the relationship between the administered cell numbers and CSF neurotrophin concentrations. CSF results were available for 245 of the 252 planned samples (42 patients × 3 cycles × 2 time points).

### 2.6. Safety Assessment

Safety endpoints were defined before the first administration and have been described previously [31]. Immediate adverse reactions comprised respiratory failure, allergic reaction, local complications, systemic complications, and paralysis or sensory loss below the level of the injection site. Delayed reactions comprised intraspinal tumour formation, syringomyelia, persistent sensory loss, or paralysis not attributable to disease progression. Patients were observed during the seven-day hospitalisation following each administration and questioned at every subsequent visit. Events were recorded descriptively. They were not graded for severity or tabulated according to a standard harm taxonomy.

Patients who stopped attending scheduled assessments were not followed with scale assessments. Vital status was available for all 42 treated patients up to week 90 and forms the basis of the survival analysis. It was obtained by telephone contact with the patients or their caregivers, not according to a standardised schedule, and no structured enquiry about adverse events was made after discontinuation. Adverse events, serious adverse events and deaths are summarised in Section 3.7.

### 2.7. Statistical Analysis

Analyses were performed with STATISTICA 14.0 (TIBCO Software Inc., Palo Alto, CA, USA). Distributions were assessed with the Shapiro–Wilk test. Because most variables departed from normality and groups were small, non-parametric methods were used throughout, and results for the clinical scales are presented as medians unless stated otherwise. Longitudinal within-group change was tested with Friedman’s ANOVA with Kendall’s coefficient of concordance, applied separately to three prespecified windows: the whole observation period (weeks 0–24), the on-treatment period (weeks 0–13) and the post-treatment period (weeks 13–24 for the Norris scale and weeks 16–24 for the ALSAQ-40, reflecting the timing of the last stable assessment for each instrument). Pairwise comparison of two dependent measurements was performed with the Wilcoxon signed-rank test, and comparison between independent strata at a given time point with the Mann–Whitney U test or Kruskal–Wallis rank ANOVA. Categorical baseline characteristics were compared with Fisher’s exact test. Associations between cell counts and CSF protein concentrations were tested with Spearman’s rank correlation. Survival was analysed by the Kaplan–Meier method with the Gehan–Wilcoxon and log-rank tests. Two-sided *p* values below 0.05 were considered significant. Because the study was exploratory, the sample size was not based on a formal power calculation, and no interim analyses or stopping rules were prespecified.

Two features of this analysis plan require emphasis. First, all repeated-measure tests were performed on complete cases. Because the number of patients contributing a full set of measurements differed between instruments, time windows and strata, the effective N of individual Friedman tests ranged from 4 to 21 and is reported alongside each result. Tests based on the smallest strata have very little power, and a non-significant result in those strata should not be read as evidence of stability. Second, no correction for multiple comparisons was applied. Across three instruments, three time windows, five strata, seven assessment points, and eight survival subgroup comparisons, approximately 100 significance tests were performed (the additional post hoc analyses increase this number), so at α = 0.05 roughly five spurious findings are expected. A Bonferroni-corrected threshold for that number of tests would be α = 0.0005. The only results that meet it are the post-treatment decline in the Norris scale among completers (*p* = 0.00004) and in two of the four strata (*p* = 0.00004 and *p* = 0.00008). The post-treatment decline in the ALSAQ-40 among completers (*p* = 0.0019), all between-stratum comparisons, and all survival comparisons do not. All subgroup and time-point comparisons are consequently exploratory and hypothesis-generating.

Post hoc strata were defined by the median-adjacent cut-off points used throughout the programme: total MNC yield < 600 × 10^6^ (stratum A) versus ≥600 × 10^6^ (stratum B), and total administered LIN^−^ cell dose < 15 × 10^6^ (stratum I) versus ≥15 × 10^6^ (stratum II). For the analysis of individual administrations, LIN^−^ strata were defined by a 5 × 10^6^ cut-off point, approximating the median dose per procedure (median 4.5 × 10^6^ at the first administration). The MNC-based and LIN^−^-based strata are not congruent and comprise different patients. They are therefore reported and interpreted separately.

#### Sensitivity and Additional Analyses

Because the longitudinal analysis was restricted to completers, we additionally analysed all 42 treated patients using the assessments available at weeks 0, 4, 6, 7, 10, 12, 13 and 16 (that is, at the start of each cycle and 7 and 28 days after each administration). Norris and ALSFRS-R scores were modelled with linear mixed models with a random intercept for each patient and time (in months) as a fixed effect, which uses all available observations under a missing-at-random assumption. Slopes are given with 95% confidence intervals (CIs), and a piecewise model with a change in slope at week 13 was fitted to describe the post-treatment period. Individual on-treatment ALSFRS-R slopes (weeks 0–13, patients with at least three assessments) were compared with the individual pre-treatment progression rate, ΔFS = (48—baseline ALSFRS-R)/disease duration in months, using the Wilcoxon signed-rank test. Baseline characteristics of completers and non-completers were compared with Welch’s *t*-test and Fisher’s exact test using summary statistics of the whole treated cohort and of the completers. Values for non-completers were derived by subtraction because completion status was not linked to the baseline database at patient level. CSF BDNF concentrations before and 7 days after each administration were compared with the Wilcoxon signed-rank test. The association between the number of LIN^−^ cells and day-7 BDNF was assessed in all patients with paired samples by Spearman correlation, by partial Spearman correlation adjusted for the day-0 concentration, and by linear regression of log-transformed day-7 BDNF on log-transformed cell number and log-transformed day-0 BDNF. All these analyses were post hoc and were not corrected for multiplicity.

## 3. Results

### 3.1. Patients, Disposition and Cell Product

Of the 45 patients qualified, 42 received all three intrathecal LIN^−^ cell administrations and 23 completed the predefined 24-week assessment schedule and constituted the longitudinal efficacy cohort (Figure 1). The 19 treated patients who did not complete the schedule discontinued because neurological and functional deterioration made attendance at scheduled visits impossible. No deaths occurred within the first 24 weeks. Baseline characteristics of the qualified cohort are given in Table 1.

The longitudinal efficacy cohort comprised 12 men and 11 women aged 27–65 years (mean 53.6 ± 10.8 years). Disease duration at inclusion ranged from 5 to 120 months (mean 31.4 ± 29.1 months); 18 patients had limb-onset and 5 bulbar-onset disease. Median baseline scores were 94 points on the Norris scale, 30 points on the ALSFRS-R and 30 points on the ALSAQ-40 total score, indicating moderate functional impairment with relatively preserved self-reported quality of life. Table 1 describes all 45 patients qualified for the study. Because the three patients who never received cells contributed no follow-up data, every analysis reported below is by definition a per-protocol analysis. No intention-to-treat analysis was possible. The sensitivity analyses in Section 3.8 include all 42 treated patients.

Characteristics of the cell product are summarised in Table 2. The yield of isolated MNCs and the number of administered LIN^−^ cells varied more than tenfold between patients and in some cases between successive procedures in the same patient. The proportion of LIN^−^ cells relative to the MNC yield (cumulative LIN^−^ dose divided by cumulative NC yield, calculated per patient) was 3.3 ± 1.6% (median 3.3%, range 0.5–6.9%). Baseline characteristics of the longitudinal cohort stratified by MNC yield and by LIN^−^ dose are shown in Table 3 and Table 4. The strata were comparable for age, disease duration, site of onset and all three clinical scales, but sex distribution differed markedly between the MNC strata: 90% of patients in stratum B were men compared with 23% in stratum A (*p* = 0.003). This imbalance is discussed below.

Completion status was not linked to the baseline database at patient level. Baseline values of the 19 non-completers were therefore derived from the summary statistics of all 42 treated patients and of the 23 completers (Table 5). Non-completers tended to have lower baseline ALSFRS-R (28.0 ± 4.2 versus 30.8 ± 5.3 points; *p* = 0.067) and Norris scores (82.3 ± 11.4 versus 90.7 ± 16.4 points; *p* = 0.061) than completers, whereas age, disease duration, cumulative MNC yield and cumulative LIN^−^ dose did not differ. With 19 and 23 patients, these comparisons are underpowered and the derived values are approximate.

The timing of loss-to-scale follow-up is shown in Table 6. Norris and ALSFRS-R scores were available for 42 patients at baseline, for 38–39 at week 4, for 40 at each assessment between weeks 6 and 13, for 34 at week 16 and for 23 at week 24. Eight patients therefore had no recorded assessment at week 16, and a further 11 were lost between weeks 16 and 24. The date of the last assessment and the interval between the last assessment and death were not recorded for these patients, and individual reasons for discontinuation were not documented in the analysed database. The investigators attributed discontinuation to functional deterioration.

### 3.2. Functional Status: Norris Scale

**Figure 2:**
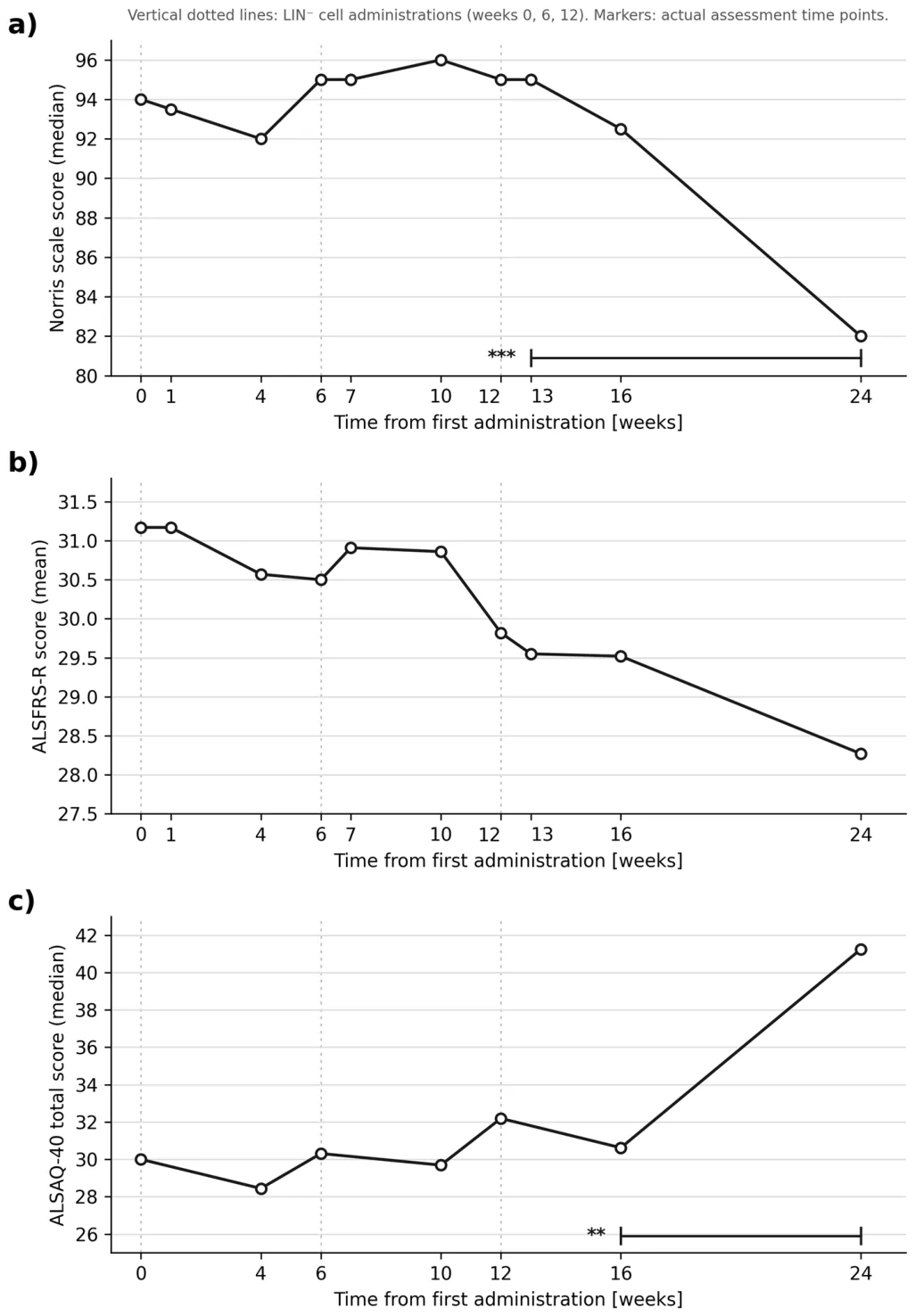
Longitudinal course of clinical and quality-of-life measures in the whole longitudinal efficacy cohort (*n* = 23). (**a**) Norris scale; (**b**) revised ALS Functional Rating Scale (ALSFRS-R); (**c**) ALSAQ-40 total score, on which higher values denote worse quality of life. Cell administrations took place at weeks 0, 6 and 12. Horizontal bars mark intervals of statistically significant within-group change (Friedman ANOVA): *** *p* < 0.0006, ** *p* < 0.005. Mn—median (arithmetic means are plotted for the ALSFRS-R, for which the median distribution was uninformative). The abscissa is proportional to time; symbols mark the actual assessment time points and dotted vertical lines the administrations (weeks 0, 6 and 12). The ALSAQ-40 was not assessed at weeks 1, 7 or 13.

Among the 23 completers, the median Norris score was 94 points at baseline and remained within a 92- to 96-point band throughout the treatment period, reaching 95 points at week 13—one week after the third administration. Between week 13 and week 24, the score fell to 82 points, a highly significant change (Friedman ANOVA, *p* = 0.00004; Figure 2a). This corresponds to a rate of decline of 5.13 points per month against a rate of −0.33 points per month (i.e., a marginal improvement) over the preceding treatment period. Over the entire 0–24-week window, the omnibus test was likewise significant, but the effect was attributable exclusively to the post-treatment interval. In the entire cohort of treated patients, however, no plateau was observed (Section 3.8).

**Figure 3:**
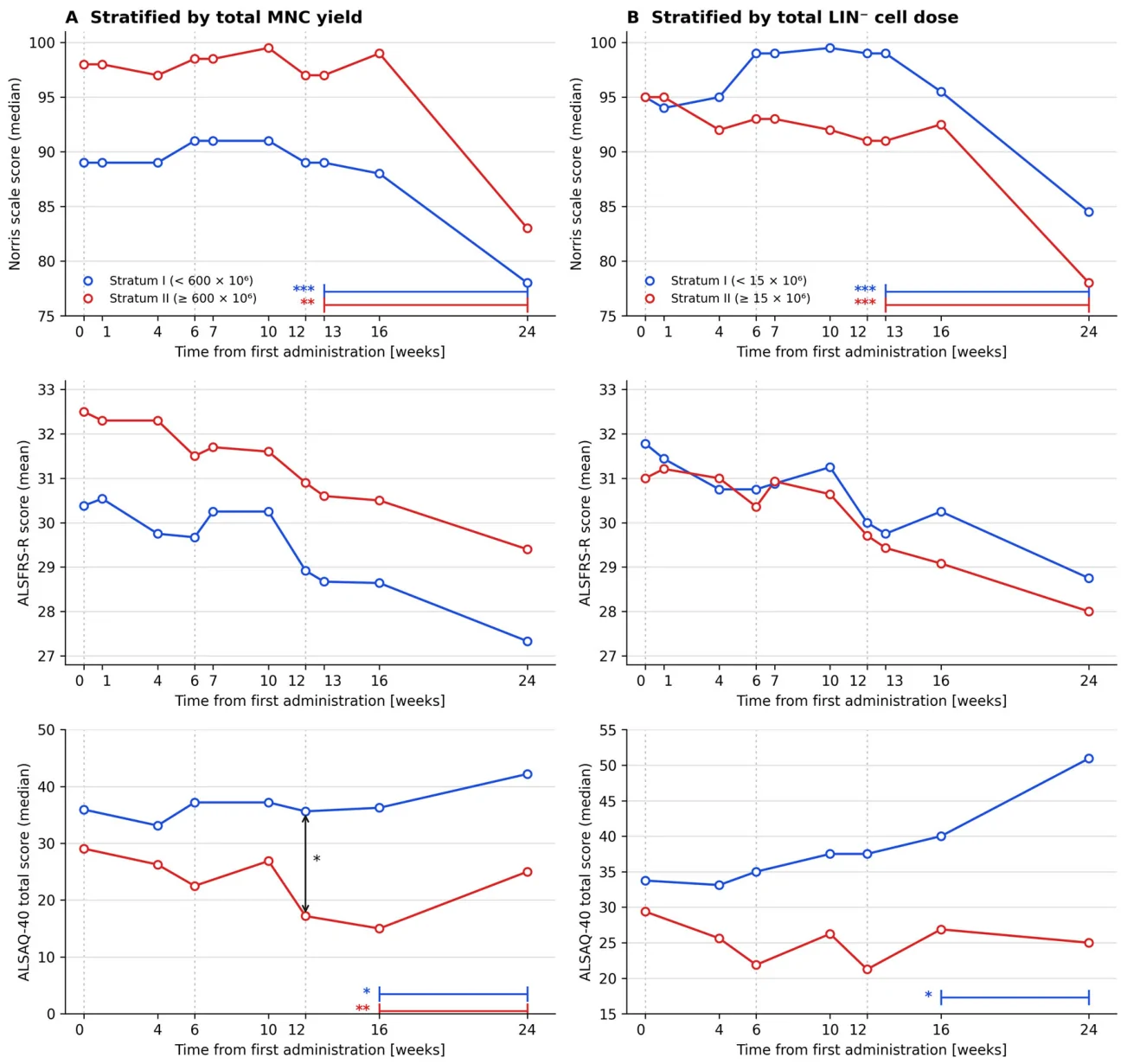
Longitudinal course of clinical and quality-of-life measures according to post hoc strata. (**A**) Patients stratified by cumulative MNC yield: strata labelled I and II in panel (**A**) correspond to strata A and B in the text; stratum I (blue) < 600 × 10^6^, *n* = 13; stratum II (red) ≥ 600 × 10^6^, *n* = 10. (**B**) Patients stratified by cumulative administered LIN^−^ cell dose: stratum I (blue) < 15 × 10^6^, *n* = 9; stratum II (red) ≥ 15 × 10^6^, *n* = 14. Rows from top to bottom: Norris scale, ALSFRS-R, ALSAQ-40 total score. Horizontal bars in the corresponding colour mark intervals of significant within-stratum change; the vertical double arrow marks a significant between-stratum difference at a single time point (Kruskal–Wallis rank ANOVA). *** *p* < 0.0006, ** *p* < 0.005, * *p* < 0.05. The MNC-based and LIN^−^-based strata are not congruent and comprise different patients. Mn—median. The abscissa is proportional to time; symbols mark the actual assessment time points and dotted vertical lines the administrations (weeks 0, 6 and 12). The ALSAQ-40 was not assessed at weeks 1, 7 or 13.

The same biphasic pattern was reproduced in every stratum (Figure 3, upper row). Among patients stratified by MNC yield, median baseline scores were 89 points in stratum A and 98 points in stratum B. Neither stratum changed significantly between baseline and week 13 (*p* = 0.076, N = 11 and *p* = 0.925, N = 10, respectively), whereas both deteriorated significantly between weeks 13 and 24 (stratum A, 89→78 points, *p* = 0.00004, N = 11; stratum B, 97→83 points, *p* = 0.00147, N = 7). Among patients stratified by LIN^−^ dose, both strata started from an identical median of 95 points. Stratum I rose to 99 points by week 6 and held this level until week 13, while stratum II remained between 91 and 93 points. Again, no significant change occurred during the treatment period (*p* = 0.456, N = 8 and *p* = 0.600, N = 13), and both strata deteriorated significantly thereafter (stratum I, 99→84.5 points, *p* = 0.00058, N = 8; stratum II, 91→78 points, *p* = 0.00008, N = 10).

No statistically significant between-stratum difference was detected at any single time point for the MNC or LIN^−^ stratification.

### 3.3. Functional Status: ALSFRS-R

The ALSFRS-R behaved differently. Mean scores fell from 31.2 points at baseline (patients with complete ALSFRS-R data, N = 21; 30.8 points in all 23 completers, Table 3) to 28.0 points at week 24 in a broadly monotonic fashion, with only shallow transient upticks after the second administration and no interval of stabilisation (Figure 2b). The omnibus Friedman test across all ten assessments was significant (χ^2^ = 66.78, N = 21, *p* < 0.001), but—in contrast to the Norris scale—no prespecified interval, including weeks 13–24, reached significance on its own.

The absence of an inflection is not merely a matter of statistical significance. The rate of decline itself was constant. Mean ALSFRS-R fell from 31.17 to 29.55 points between baseline and week 13, that is, by 0.54 points per month, and from 29.55 to 28.27 points between weeks 13 and 24, that is, by 0.50 points per month. The two windows are therefore indistinguishable, in sharp contrast to the fifteenfold change in the rate of decline of the Norris scale over the same intervals. Moreover, the pre-treatment rate of progression estimated by the conventional method—(48—baseline ALSFRS-R) divided by symptom duration—was 0.55 points per month when calculated from the mean baseline score and mean disease duration of this cohort (the median of individually calculated values in all treated patients was 0.84 points per month; Section 3.8), which is essentially identical to the 0.52 points per month observed over the whole 24-week study period. On this instrument, therefore, patients declined at the same rate before, during and after the treatment course. The implications of this discordance are considered in Section 4.3. Because this comparison rests on group means and on a pre-treatment rate estimated from a single time point, it is descriptive. Individual-level analyses in all treated patients are given in Section 3.8.

Stratification did not alter this picture (Figure 3, middle row). By MNC yield, stratum B started higher and remained higher throughout (32.5→29.4 points) than stratum A (30.4→27.3 points), yet the magnitude of decline was virtually identical (−3.1 vs. −3.0 points). By LIN^−^ dose, stratum I fell from 31.8 to 28.8 points and stratum II from 31.0 to 28.0 points (−3.0 points in both). No between-stratum difference reached significance at any time point, and neither the cumulative MNC yield nor the cumulative LIN^−^ dose correlated with the rate of ALSFRS-R progression.

The total decline of approximately 3 points over 24 weeks corresponds to roughly 0.55 points per month, which is at the slower end of but well within the range reported for untreated ALS populations. This estimate refers to completers, who were selected for slower decline. In all treated patients, the ALSFRS-R declined by 0.72–0.75 points per month up to week 16 (Section 3.8).

### 3.4. Health-Related Quality of Life: ALSAQ-40

Among the 23 completers, the median ALSAQ-40 total score was 30.0 points at baseline. It improved slightly four weeks after the first administration (28.4 points) and then oscillated within a narrow band through week 16 (30.3, 29.7, 32.2 and 30.6 points at weeks 6, 10, 12 and 16, respectively). Between week 16 and week 24, quality of life deteriorated abruptly to 41.3 points (*p* = 0.0019; Figure 2c). The instrument thus reproduced the biphasic pattern seen with the Norris scale, but with the inflection displaced by approximately three weeks, consistent with the expected lag between loss of motor function and its subjective consequences.

Stratification by MNC yield (Figure 3, lower left). Patients in stratum B, in whom a higher marrow yield had been obtained, improved slightly over the whole observation period (29.1→25.0 points), whereas patients in stratum A deteriorated (35.9→42.2 points). Both strata worsened significantly between weeks 16 and 24 (stratum A *p* = 0.028; stratum B *p* = 0.0028). Between-stratum comparison reached significance only at week 12 (35.6 vs. 17.2 points, Kruskal–Wallis *p* = 0.034), with non-significant trends in the same direction at week 16 (36.3 vs. 15.0 points, *p* = 0.070) and week 24 (42.2 vs. 25.0 points, *p* = 0.063).

Stratification by LIN^−^ dose (Figure 3, lower right). Patients in stratum II, who received a cumulative dose ≥ 15 × 10^6^ LIN^−^ cells, ended the observation period slightly better than they began it (29.4 points at baseline, 25.0 points at week 24; change −4.4 points), whereas stratum I deteriorated progressively (33.8→50.9 points; change +17.2 points). Comparison of absolute scores showed a difference in favour of stratum II at week 4 (33.1 vs. 25.6 points, *p* = 0.028), week 12 (37.5 vs. 21.3 points, *p* = 0.02), week 16 (40.0 vs. 26.9 points, *p* = 0.004) and week 24 (50.9 vs. 25.0 points, *p* = 0.03).

These between-stratum comparisons must however be read against the baseline: stratum II already reported a better total score before any cells were given (29.4 vs. 33.8 points) and a significantly better communication domain score (35.7 vs. 78.6 points, *p* = 0.02). Comparisons of absolute values at successive visits cannot separate a treatment-related divergence from the persistence of that initial difference, and patient-level change scores were not available for a formal analysis of change from baseline. Within-stratum tests are not vulnerable to the same objection, but they are severely underpowered: over the whole observation period the ALSAQ-40 total score worsened significantly in stratum I (Friedman *p* = 0.0136) but not in stratum II (*p* = 0.198), yet these tests included only six and four patients with complete data, respectively, so the non-significant result in stratum II carries almost no information. Within stratum I the deterioration between weeks 16 and 24 was also significant (*p* = 0.028). Taken together, the direction of the dose-related difference is consistent across every analysis, but the present data cannot establish that it is attributable to the treatment.

Domain-level analyses were consistent with the total score, but must be interpreted with particular caution given the number of comparisons. The advantage of stratum II was carried mainly by the eating and drinking, communication and emotional reactions domains, whereas the physical mobility and activities of daily-living domains showed no between-stratum difference and declined in parallel in both. Importantly, stratum II already reported better communication scores at baseline (35.7 vs. 78.6 points, *p* = 0.02), so the domain-level findings cannot be attributed to treatment on the basis of the present design.

### 3.5. Survival

**Figure 4:**
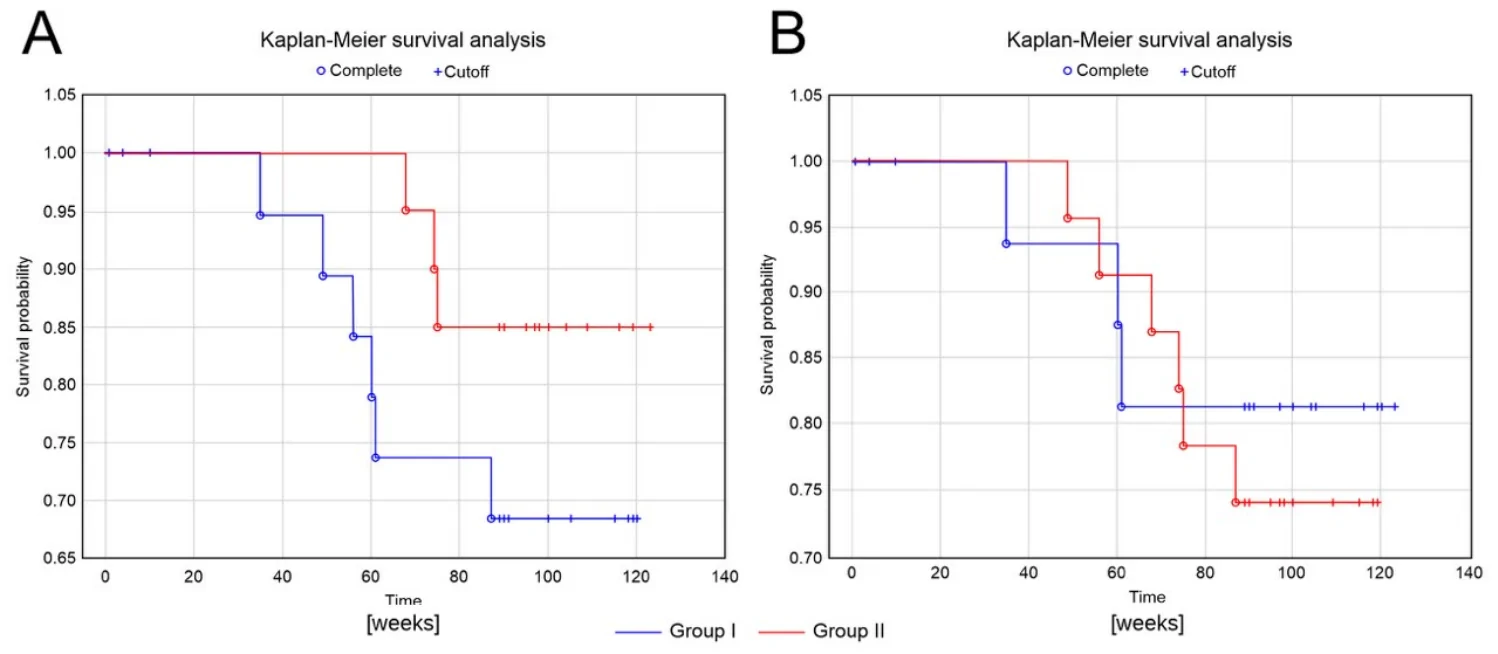
Kaplan–Meier survival estimates in all treated patients (*n* = 42), with observations censored at week 90. (**A**) Patients stratified by cumulative MNC yield (stratum I < 600 × 10^6^, stratum II ≥ 600 × 10^6^); Gehan–Wilcoxon *p* = 0.156, log-rank *p* = 0.186. Strata I and II in panel A correspond to strata A and B in the text. (**B**) Patients stratified by cumulative administered LIN^−^ cell dose (stratum I < 15 × 10^6^, stratum II ≥ 15 × 10^6^); Gehan–Wilcoxon *p* = 0.750, log-rank *p* = 0.669. Circles denote deaths and tick marks censored observations.

Survival analyses are secondary and exploratory: only nine events occurred, ascertainment of vital status was not standardised, and strata were defined post hoc. Nine deaths occurred among the 42 treated patients within the 90-week observation window used for the survival analysis. Kaplan–Meier curves stratified by cumulative MNC yield (Figure 4A) separated visibly in favour of stratum B from week 35 onwards, with a survival probability of 0.85 versus 0.68 at the end of observation. The difference did not reach significance (Gehan–Wilcoxon *p* = 0.156; log-rank *p* = 0.186).

Stratification by cumulative LIN^−^ dose produced no separation of the survival curves (Gehan–Wilcoxon *p* = 0.750; log-rank *p* = 0.669; Figure 4B).

In an exploratory analysis of individual administrations, none of the six comparisons based on MNC yield or LIN^−^ dose at the second or third procedure approached significance (*p* ≥ 0.155). The single exception was the dose administered at the first procedure: patients who received ≥ 5 × 10^6^ LIN^−^ cells at the first administration (*n* = 19) showed a longer time to death than those who received less (*n* = 23), with a survival probability at the end of observation of 0.94 versus 0.65 (Gehan–Wilcoxon *p* = 0.033; log-rank *p* = 0.035; Figure S1). Eight of the nine deaths occurred in the lower-first-dose subgroup, and the higher-first-dose subgroup contributed a single death, at week 75. A survival comparison in which one arm contributes one event is not informative on its own. This was moreover one of eight uncorrected subgroup comparisons. It does not meet the corrected significance threshold defined in Section 2.7, and neither the second nor the third administration reproduced it. The result is therefore presented as Supplementary Material and must be regarded as hypothesis-generating only.

### 3.6. Cerebrospinal Fluid Neurotrophins in Relation to Cell Dose

Consistent with the detailed humoral analyses published previously from this programme [31,32,33,34], CSF concentrations of BDNF, NGF-β, NT-3, TNF-α, TNF-R and CRP showed no consistent statistically significant change across the three treatment cycles, and no significant differences between the MNC- or LIN^−^-defined strata at any time point. The full biomarker dataset is therefore not reproduced here.

**Figure 5:**
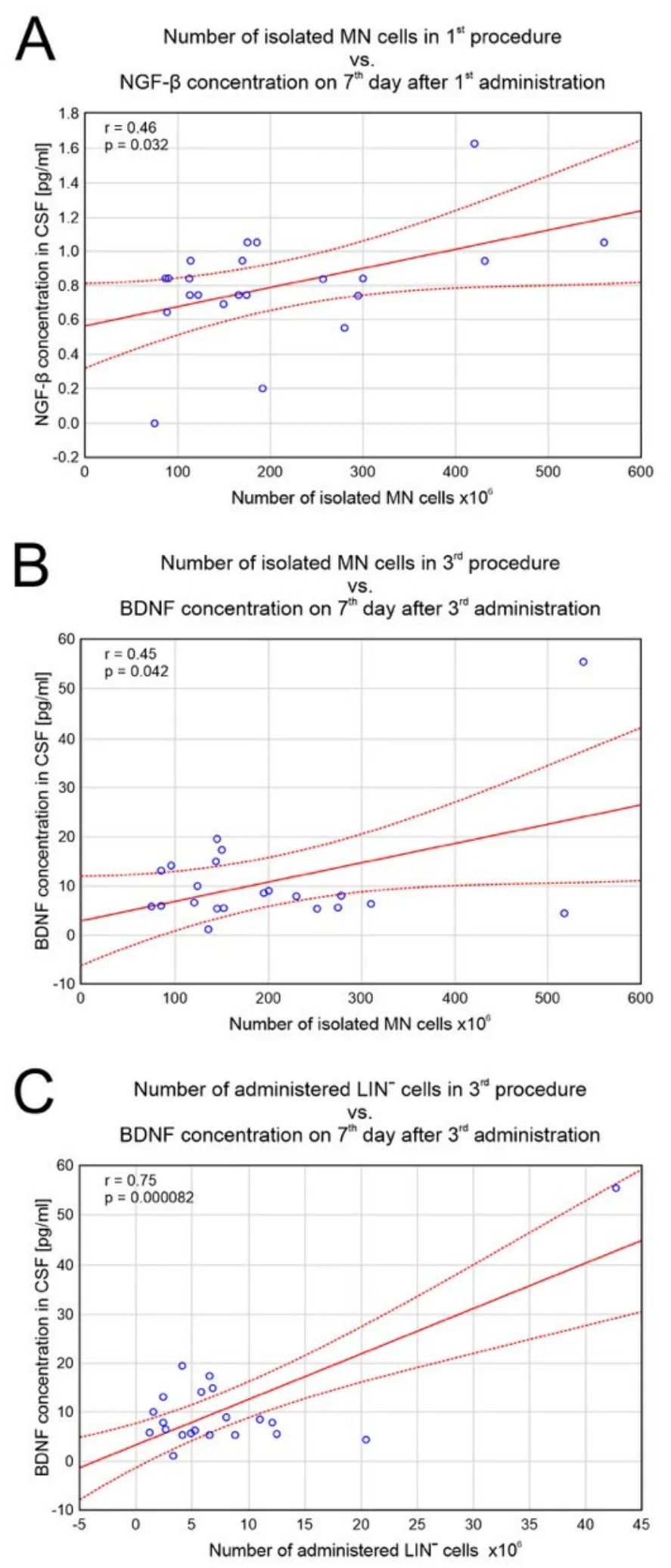
Spearman rank correlations between the amount of cellular material and cerebrospinal fluid neurotrophin concentrations on day 7 of the corresponding treatment cycle. (**A**) MNC yield of the first procedure versus NGF-β after the first administration (r = 0.46, *p* = 0.032). (**B**) MNC yield of the third procedure versus BDNF after the third administration (r = 0.45, *p* = 0.042). (**C**) Number of LIN^—^ cells administered at the third procedure versus BDNF after the third administration (r = 0.75, *p* = 0.000082). Solid lines represent the regression fit and dashed lines the 95% confidence interval. All three correlations were calculated in completers with available samples (patients with complete follow-up; *n* = 22 in panel (**C**)); it was not reproduced in all 39 patients with paired samples (ρ = 0.14, *p* = 0.39; Section 3.6).

Three positive correlations between the amount of cellular material and the CSF response did however reach significance (Figure 5). The MNC yield of the first procedure correlated with the NGF-β concentration on day 7 after the first administration (r = 0.46, *p* = 0.032); the MNC yield of the third procedure correlated with the day-7 BDNF concentration after the third administration (r = 0.45, *p* = 0.042); and most strongly, the number of LIN^−^ cells administered at the third procedure correlated with the day-7 BDNF concentration after that administration (r = 0.75, *p* = 0.000082) in the 22 completers with available day-7 samples, i.e., the patients with complete follow-up in whom the correlation analyses were originally performed. The correlations in Figure 5A,B were likewise calculated in completers with available samples. They were not re-examined in the full sample, and—like those in Figure 5C—should be regarded as unconfirmed. When all 39 patients with paired BDNF samples in cycle III were analysed, the number of LIN^−^ cells did not correlate with day-7 BDNF (Spearman ρ = 0.14, *p* = 0.39) or day-0 BDNF (ρ = −0.14, *p* = 0.38), the partial correlation adjusted for day-0 BDNF was weak (ρ = 0.28, *p* = 0.09), and after exclusion of the three highest BDNF values the association vanished (ρ = −0.05). The association shown in Figure 5C is therefore not robust. Within-cycle changes in BDNF (day 0 to day 7) were not significant in any cycle (Wilcoxon *p* = 0.51, 0.65 and 0.15 for cycles I–III; Table S2), whereas the BDNF concentration before the third administration was higher than before the first (median difference 1.98 pg/mL, *p* = 0.0009). The within-cycle rise in BDNF in cycle III correlated modestly with the number of LIN^−^ cells (ρ = 0.40, *p* = 0.012), and in a log–log regression adjusted for day-0 BDNF, the coefficient for cell number was 0.27 (95% CI −0.01 to 0.55, *p* = 0.058; Table S3). Taken together, these data do not allow a conclusion that the number of administered cells determines the neurotrophic response within the CNS compartment.

### 3.7. Safety

Safety was a primary outcome of this programme from its inception and was assessed against criteria defined before the first administration [31]. Immediate adverse reactions were prespecified as respiratory failure, allergic reaction, local complications, systemic complications, and paralysis or sensory loss below the level of the injection site. Delayed reactions were prespecified as intraspinal tumour formation, syringomyelia, persistent sensory loss, or paralysis not attributable to progression of the disease. Patients were monitored throughout the seven-day hospitalisation that followed each administration and at every subsequent scheduled visit.

None of these events occurred. Across the 126 bone marrow aspirations and 126 intrathecal administrations performed in the 42 treated patients—each cell administration being accompanied by a second, diagnostic lumbar puncture on day 7—no immediate or delayed, local or systemic adverse event attributable to the harvest or to the intrathecal procedure was recorded. This is consistent with what has been reported for successive subsets of the same cohort, both after a single administration [31,32,35,36] and after the full course of three administrations [33,34,37].

Two procedural precautions were applied throughout and are worth restating, because both were introduced specifically to limit harm. The cell suspension was injected slowly, over 2–3 min, and patients remained supine for 24–48 h after each procedure in order to reduce the risk of post-dural puncture headache [31,33]. In addition, the number of lumbar punctures per treatment cycle was deliberately restricted to two—on the day of administration and on day 7—even though a denser sampling schedule would have been informative, because repeated dural puncture in a population with progressive respiratory compromise was judged ethically unacceptable [34].

A structured summary of procedures, recorded events and completeness of follow-up is provided in Table 7. Because the trial was designed and conducted before structured harm reporting became standard practice in this setting, adverse events were captured descriptively during hospitalisation and follow-up rather than graded by system organ class. Table 7 therefore documents what was and was not systematically ascertained and is not an adjudicated event count. The statement above should be read as the absence of any clinically apparent complication over 126 procedures in 42 patients, not as a formally adjudicated harm analysis. This constraint is acknowledged among the limitations.

### 3.8. Sensitivity Analyses in All Treated Patients

Because the longitudinal analysis above was restricted to the 23 completers, all 42 treated patients were analysed using all available observations up to week 16 (post hoc; Table 6). In a random-intercept linear mixed model, the Norris score declined by 0.95 points per month between baseline and week 13 (95% CI 0.52–1.38) and by 1.19 points per month between baseline and week 16 (95% CI 0.82–1.57). Generalised estimating equations gave identical point estimates with wider intervals (0.95 points per month, 95% CI 0.24–1.66, for weeks 0–13). A piecewise model with a change in slope at week 13 gave an on-treatment decline of 0.95 points per month (95% CI 0.50–1.39) and an additional decline of 2.36 points per month thereafter (95% CI 0.04–4.67; *p* = 0.046; scores after week 16 are available only for completers). In the entire treated cohort, the Norris score therefore fell during the treatment period, and the plateau observed in completers was not reproduced.

The ALSFRS-R declined by 0.75 points per month up to week 13 (95% CI 0.59–0.91) and by 0.72 points per month up to week 16 (95% CI 0.58–0.85). The change in slope after week 13 was +0.31 points per month (95% CI −0.52 to 1.13; *p* = 0.468), providing no evidence of a change in the rate of decline.

Individual ALSFRS-R slopes between weeks 0 and 13 (40 patients with at least three assessments) showed a median on-treatment decline of 0.71 points per month (interquartile range 0.00–1.38; mean 0.75) compared with a median estimated pre-treatment rate ΔFS of 0.84 points per month (interquartile range 0.50–1.66; mean 1.11) [46]. The difference was not significant (Wilcoxon signed-rank test, *p* = 0.20). Because ΔFS assumes linear decline from a full score at symptom onset and individual on-treatment slopes are estimated from at most seven assessments, this comparison is imprecise and cannot distinguish a treatment effect from measurement variability.

## 4. Discussions

### 4.1. Principal Findings

This report describes the clinical and patient-reported outcomes of a programme of three intrathecal administrations of autologous bone marrow-derived LIN^−^ cells in sporadic ALS, followed for 24 weeks with survival ascertainment to week 90. Three findings deserve emphasis, and each carries an important qualification. First, two of the three instruments used—the Norris scale and the ALSAQ-40—showed a plateau lasting from baseline to the end of the treatment period, followed by significant deterioration once treatment stopped in the 23 patients who completed the assessment schedule, whereas in all 42 treated patients the Norris score declined during treatment (0.95 points per month; Section 3.8). The third instrument, the ALSFRS-R, showed no such inflection and declined at a constant rate that was also indistinguishable from the estimated pre-treatment rate. Second, quality of life diverged consistently according to the number of LIN^−^ cells administered, but the higher-dose stratum began the study with better scores, so the divergence cannot be attributed to treatment on the present design. Cell dose was related neither to functional decline nor to survival. Third, the number of administered cells correlated with the CSF concentration of BDNF seven days after the third procedure in a subset of 22 patients (r = 0.75), but not in all 39 patients with paired samples (ρ = 0.14), so a biological dose effect remains unproven. Only the post-treatment decline in the Norris scale survives correction for the number of comparisons performed.

### 4.2. Interpreting the Plateau

The absence of a control group means that the temporal pattern observed in completers cannot be converted into a causal claim. ALS progression is notoriously non-linear at the individual level, and a group of patients selected for their ability to attend all scheduled visits is by construction enriched for slower progressors. We report this pattern for completeness—it is not evidence of efficacy.

A simpler explanation for the plateau in completers is selection. In sum, 19 of the 42 treated patients (45%) were lost to scale follow-up before week 24. Their baseline Norris and ALSFRS-R scores tended to be lower than those of completers (Table 5), and in the whole treated cohort the Norris score fell by 0.95 points per month during treatment (Section 3.8). Analyses restricted to patients who remain under observation overestimate stability whenever faster progressors drop out, and informative attrition is a recognised source of bias in ALS trial data sets [47]. Long-lasting plateaus and reversals of functional decline are uncommon in large ALS trial populations [48], so a plateau lasting three months in a group of this size is more plausibly explained by selection and by the measurement properties of the Norris scale than by a biological arrest of the disease.

### 4.3. Why the Norris Scale and the ALSFRS-R Diverged

The discordance between the instruments is the central interpretative problem of this study, and we prefer to state it in full rather than in summary. Among the 23 completers, over the treatment period the Norris scale improved marginally, at −0.33 points per month, and then fell at 5.13 points per month after treatment stopped—a fifteenfold change in rate. The ALSAQ-40 behaved similarly, worsening at 0.17 points per month to week 16 and at 5.77 points per month thereafter. The ALSFRS-R did neither: it fell at 0.54 points per month before week 13 and at 0.50 points per month afterwards, and the rate observed across the whole study, 0.52 points per month, is close to the 0.55 points per month estimated from the cohort’s mean baseline score and disease duration. On the instrument accepted as the standard measure of disease progression in ALS, nothing changed. In all 42 treated patients, by contrast, both instruments declined during treatment (Norris 0.95 and ALSFRS-R 0.75 points per month), so the discordance is confined to completers.

Two explanations compete, and the present design cannot separate them.

The first is clinimetric. The ALSFRS-R is a 12-item, 48-point ordinal scale in which a one-point change requires a categorical change in an activity of daily living; the Norris scale distributes 21 items across a wider numerical range, is administered by physical examination rather than by interview, and weights bulbar and fine motor function more heavily. A coarse instrument will register a smaller effect than a fine one, and a trial powered on the ALSFRS-R alone may be blind to changes that a finer-grained or patient-reported measure can detect—an argument also made in relation to recent phase III cell therapy programmes, in which prespecified subgroups showed benefit that the primary endpoint in the whole population did not capture [27,28,29]. The clinimetric argument is weakened however by the magnitude of what it must explain: coarse granularity attenuates the size of an effect, but it does not abolish a fifteenfold difference in the rate of change.

The second explanation is that the two instruments that moved are precisely the two most vulnerable to unblinding. The Norris scale was administered by an unblinded investigator, and the ALSAQ-40 is a self-report questionnaire completed by patients who knew they were receiving an experimental cell therapy and who were hospitalised for seven days after each administration. The ALSFRS-R, although also collected openly, is anchored to concrete functional milestones—whether the patient walks unaided, needs a gastrostomy, requires ventilatory support—which are considerably harder to shift by expectation. The distribution of the effect across the three instruments is exactly what an expectation effect would produce, and it coincides in time with an intensive, visible intervention. We cannot exclude it, and we do not think it should be excluded on the authors’ say-so. A further, non-exclusive explanation is selection: completers had somewhat better baseline scores and were by definition able to attend later visits (Section 3.1), and the whole-cohort analysis showed no plateau.

The clinimetric reading can be tested and the expectation reading can be controlled for, but neither can be settled here. Item-level analysis of the Norris scale, showing whether the plateau is carried by the bulbar and fine motor items that the ALSFRS-R covers only coarsely, would support the first explanation. A sham-controlled design with blinded raters would eliminate the second. Both belong in the next trial, and we regard this discordance as the principal reason why the present findings cannot be presented as evidence of efficacy.

### 4.4. Quality of Life and the Dose Signal

The most consistent dose-related pattern in this study concerned quality of life, and it is also the finding most exposed to confounding. Patients receiving a cumulative dose of at least 15 × 10^6^ LIN^−^ cells ended the observation period 4.4 points better than they began it, whereas those receiving less deteriorated by 17.2 points, and comparison of absolute scores favoured the higher-dose stratum at four of seven time points. Three caveats are decisive rather than incidental. Stratum II began the study with better quality of life (29.4 vs. 33.8 points) and with significantly better communication scores, so comparison of absolute values at successive visits cannot distinguish a treatment-related divergence from the persistence of that initial difference. Patient-level change scores were not available to us for a formal analysis of change from baseline, and this analysis should be regarded as outstanding. The within-stratum tests, which are immune to that objection, included only six and four patients with complete data, so the absence of significant deterioration in the higher-dose stratum cannot be read as evidence of stability. Also, the strata were defined post hoc on a variable—the number of cells successfully isolated—that is itself a function of patient biology. What can be said is that the direction of the difference was the same in every analysis we performed. What cannot be said is that the treatment produced it.

Descriptively, the advantage of the higher-dose stratum was carried by eating and drinking, communication and emotional reactions, not by physical mobility or activities of daily living, which declined in parallel in both strata. This pattern is compatible with our earlier observation that LIN^−^ cell administration was followed by measurable improvement in articulatory and phonatory function in a subset of patients [35,36,37]. It rests, however, on four to six patients per stratum with complete data and on a stratum that already had better communication scores at baseline. It is also consistent with the well-described dissociation between physical impairment and self-reported well-being in ALS, in which emotional and communicative domains follow a trajectory of their own and are subject to early response shift [49,50]. Whether a bulbar and affective signal of this kind is a genuine treatment effect, an expectation effect amplified by an intensive and visible intervention, or an artefact of baseline imbalance cannot be resolved by the present design. Given the discordance described in Section 4.3, the second possibility deserves at least equal weight with the first.

### 4.5. Cell Yield, Cell Dose and the Limits of a Quantity-Based Model

We deliberately examined two different quantities. The number of administered LIN^−^ cells is a genuine exposure. The MNC yield is not: MNCs were never given to patients, and the yield reflects marrow cellularity, which is in turn influenced by age, aspirate quality, hydration and prior myelotoxic exposure. Any association between MNC yield and outcome must therefore be read as prognostic rather than therapeutic—as a marker of host biological reserve. Seen in that light, the non-significant separation of the Kaplan–Meier curves in favour of patients with a higher MNC yield (log-rank *p* = 0.186) is best interpreted as a marker of general biological robustness rather than as an effect of the intervention. Two considerations make this more than a formality. The strata differed markedly in sex composition—90% men in stratum B against 23% in stratum A (*p* = 0.003)—and sex is itself associated with phenotype and survival in ALS. With 23 patients, this cannot be adjusted for. Any survival comparison based on MNC yield is therefore confounded by design, and we present it as a prognostic observation about the patients rather than as a dose–response observation about the therapy. Marrow cellularity may nonetheless deserve prospective study in its own right: if it indexes host reserve, it is cheap to measure and available before any cells are given.

For the true exposure—administered LIN^−^ cells—the picture was mixed: an association with quality of life that was confounded by baseline imbalance, none with the functional scales, none with survival, and an inconsistent association with the CSF BDNF response (present in a subset of 22 patients but not in all 39 patients with paired samples). Taken together, these observations do not support a simple dose–response relationship. Whether the quality of the graft rather than its size is limiting remains a hypothesis that the present data cannot test. Bone marrow varies considerably between patients in ways that a cell count does not capture: two patients yielding the same number of LIN^−^ cells may deliver very different secretomes. Our earlier finding that a higher baseline BDNF concentration identified patients with a more favourable clinical course [32,33] is compatible with this hypothesis. Future protocols should therefore incorporate potency assays—secretome profiling, viability and colony-forming capacity—rather than relying on cell number alone as a release criterion.

### 4.6. Comparison with the International Experience

Our findings should be read against the current cell therapy literature in ALS. Early studies established feasibility and safety of intraspinal and intrathecal delivery of autologous bone marrow-derived cells without demonstrating efficacy [20,22,23]. Mesenchymal stromal cell products advanced further: repeated intrathecal injections of autologous bone marrow MSCs produced a clinical benefit lasting at least six months in a randomised phase II trial, with multiple injections outperforming a single one [25], and a subsequent propensity-score-matched surveillance study of lenzumestrocel reported a significant survival advantage over an external control [30]. The phase III experience has been more sobering. The NurOwn (MSC-NTF) trial did not meet its primary endpoint in the whole population, but showed preservation of function in the prespecified subgroup with a baseline ALSFRS-R ≥ 35, together with significant changes in CSF biomarkers of neuroinflammation and neurotrophic support [27]. The ALSUMMIT phase III trial of lenzumestrocel likewise failed on its combined function-and-survival primary endpoint, yet demonstrated benefit in function, survival, respiratory capacity and neurofilament light chain in slow progressors [28,29]. A phase II study of adipose-derived MSCs reported a similar pattern, with a subset of patients progressing at least 25% more slowly after treatment [26].

The isolated survival difference according to the dose given at the first procedure (Figure S1) rests on a single death in one arm, was not reproduced for the second or third procedure, and does not meet the corrected significance threshold. It is most plausibly a chance finding and is not interpreted further.

Two lessons recur across these programmes and are directly relevant to our data. The first is that treatment effects, where present, are confined to patients with less advanced or more slowly progressing disease—a group our own inclusion criteria only partially selected for. The second is that benefit appears to require continued administration. Whether continued administration would alter the course of the disease cannot be inferred from the present data, because no plateau was present in the whole treated cohort and no dose–response relationship was demonstrated. This is a question for a controlled trial to address.

No individual-level control group was available for the present study. As an approximate benchmark, the ALSFRS-R decline observed here (0.5–0.75 points per month) lies within the range generally reported for untreated ALS populations, including the pooled placebo-arm data of the PRO-ACT database [47], in which transient plateaus in individual patients are well described [48]. To our knowledge, the Norris scale and the ALSAQ-40 are not systematically captured in that database, so a matched external comparison for these instruments was not possible. We did not attempt an unmatched historical comparison because differences in eligibility criteria, disease duration and assessment schedules would render it uninterpretable. The estimated pre-treatment progression rate ΔFS [46] provides an internal benchmark: in the whole cohort, the median on-treatment ALSFRS-R decline (0.71 points per month) did not differ significantly from the median ΔFS (0.84 points per month; *p* = 0.20; Section 3.8), i.e., no slowing of decline relative to the estimated pre-treatment rate was demonstrated.

### 4.7. Neurotrophic Factors

The humoral and immunological consequences of LIN^−^ cell administration in this cohort have been reported in detail elsewhere and are not duplicated here [31,32,33,34]. The one aspect that is new—and that motivated the present analysis—is the relationship between the amount of cellular material and the CSF response. The correlation between administered LIN^−^ cells and day-7 BDNF after the third procedure (r = 0.75, *p* < 0.001) was the strongest quantitative link between exposure and CNS biology in the subset originally analysed (*n* = 22), but it was not reproduced in all 39 patients with paired samples (ρ = 0.14, *p* = 0.39), disappeared after exclusion of the three highest values (ρ = −0.05), and was weaker after adjustment for the day-0 concentration (partial ρ = 0.28, *p* = 0.09). The rise in pre-administration BDNF at the start of the third cycle (*p* = 0.0009) and the modest correlation between cell number and the within-cycle change in BDNF (ρ = 0.40, *p* = 0.012) are compatible with a dose-related trophic signal, but do not establish it. If such a signal exists, the absence of a proportional clinical effect would suggest that the limiting step lies downstream of trophin delivery—in the half-life of secreted factors, in the receptiveness of the degenerating motor neuron, or in the breadth of the trophic repertoire assayed. The last of these is testable: our panel was restricted to three neurotrophins and three inflammatory markers, and an unbiased secretome analysis, analogous to the unbiased microRNA screening we have advocated previously [31], might identify mediators that a targeted panel misses. A further limitation of the biomarker programme is the absence of neurofilament light-chain measurements and of any measure of residual cells in the CSF, so neither neuroaxonal injury nor persistence of the administered cells could be assessed. CSF samples were not stored, so these measurements cannot be added retrospectively.

### 4.8. Limitations

Several limitations must be acknowledged. The study was open-label, non-randomised and single-centre, and included no placebo or untreated control arm. Selection and observation bias cannot be excluded, and the temporal association between treatment and clinical stabilisation cannot be interpreted causally. The longitudinal efficacy cohort was small (*n* = 23) and was defined by completion of the assessment schedule, which introduces an informative-censoring bias favouring slower progressors. The 19 treated patients who could not attend later visits had lower baseline scores (Table 5), and no plateau was seen in any of the whole treated cohort (Section 3.8). The plateau observed in completers therefore cannot be generalised to all treated patients. All subgroup analyses were post hoc, based on arbitrary, though median-adjacent cut-off points. Approximately 100 significance tests were performed without correction, so roughly five spurious findings are expected by chance, and only the post-treatment decline in the Norris scale meets a Bonferroni-corrected threshold. Every other positive result in this paper, including the quality-of-life and survival comparisons, falls below it. Repeated-measure tests were performed on complete cases and the effective N ranged from 4 to 21, so several non-significant results reflect absent power rather than absent change. No comparison of each patient’s on-study rate of decline with his or her own documented pre-treatment rate was possible, because serial scale measurements from the pre-treatment observation period were not available. Such an analysis, standard in uncontrolled cell therapy studies in ALS, would have been the most informative internal control available, and its absence is a major analytical gap. We approximated it with the single-time-point estimate ΔFS in a post hoc analysis (Section 3.8), which is imprecise because it assumes linear decline from a full score at onset. The comparison of completers with non-completers (Table 5) relied on values derived from summary statistics and is underpowered. Baseline differences between strata—most notably the sex imbalance in the MNC stratification and the better baseline quality of life in the higher-LIN^−^-dose stratum—are potential confounders that a study of this size cannot adjust for. Molecular analyses were confined to six preselected CSF proteins and did not include assessment of the functional properties, viability or secretome of the administered cells. The trial predates the routine use of neurofilament light chain as a pharmacodynamic biomarker, and the study was not designed or reported prospectively according to the CONSORT checklist, which limits comparability with contemporary trials; the present report has been prepared retrospectively in accordance with the TREND statement for non-randomised evaluations [51], and the completed checklist is provided as Table S1. For the same reason, the safety data are qualitative: adverse events were recorded descriptively rather than graded and tabulated, so the absence of complications over 126 procedures (Section 3.7) is a clinical observation and not a formally adjudicated harm analysis. Further limitations concern the characterisation of participants and of the product. Sporadic ALS was defined clinically, without systematic genetic testing, a standardised laboratory panel for the exclusion of ALS mimics beyond electrophysiology and MRI, or a dedicated assessment of upper and lower motor neuron involvement, and cognitive and behavioural function was not assessed. Release criteria and viability, sterility and endotoxin results of individual products were not available, and no potency assay was performed. Harms were not graded systematically, adverse events after discontinuation were not actively sought, and vital status was ascertained by telephone contact with patients or caregivers rather than by a standardised procedure (Table 7). No ALSAQ-40 change scores or covariate-adjusted (for example, baseline-adjusted) between-stratum comparisons were calculated, so the quality-of-life differences between dose strata are descriptive. The association between cell dose and CSF BDNF was not reproducible in the whole cohort. Finally, the sensitivity analyses in Section 3.8 were post hoc, assume that data are missing at random, and were not corrected for multiplicity.

### 4.9. Implications for Future Studies

The present data support a specific and testable design. A future trial should be randomised and sham-controlled, with blinded raters—the single most important design feature, given the discordance described in Section 4.3. It should enrol patients with less advanced disease and a documented pre-treatment progression rate, so that each patient can serve as his or her own control. It should test whether administrations need to continue beyond three. It should use a fine-grained functional instrument and a patient-reported measure alongside the ALSFRS-R, and should prespecify item-level analysis of the finer instrument. It should incorporate neurofilament light chain and an unbiased CSF secretome analysis, and should characterise the potency, not merely the number, of the administered cells. Sample size should be set for the number of events rather than the number of patients, given how little the present survival analysis could resolve with nine deaths. Reporting should follow the CONSORT statement from the abstract onwards, including a structured account of harms. Enrolment should require genetic testing, a standard work-up to exclude ALS mimics, quantification of upper and lower motor neuron involvement, and cognitive screening. The trial should prespecify the handling of missing data (for example, joint modelling of longitudinal scores and survival), record dates and reasons of discontinuation, and grade adverse events (for example with CTCAE) and follow them after discontinuation.

## 5. Conclusions

Three intrathecal administrations of autologous bone marrow-derived LIN^−^ cells at six-week intervals were feasible in patients with sporadic ALS, and no adverse event was recorded across 126 procedures, although harms were not graded systematically. In the 23 patients who completed the 24-week schedule, the Norris scale and the ALSAQ-40 were stable until week 13 and then deteriorated, whereas the ALSFRS-R declined at a constant rate. In all 42 treated patients, no plateau was present, and completers had less severe baseline disease. Quality-of-life differences by cell dose were confounded by baseline imbalance, survival analyses were exploratory, and the association between cell number and CSF BDNF was not reproducible in any of the whole cohort. These findings do not establish efficacy. A randomised, sham-controlled trial with blinded raters, documented pre-treatment progression, systematic genetic and cognitive characterisation, characterised cell potency, continued dosing, and graded safety reporting is required.

## Figures and Tables

**Table 1:** Baseline characteristics of patients with sporadic ALS qualified for the study.

Variable	Qualified Cohort (*n* = 45)
Age, years (mean ± SD)	53.76 ± 9.46
Female sex, n (%)	21 (46.7)
Male sex, n (%)	24 (53.3)
Disease duration, months (mean ± SD)	29.60 ± 25.99
Limb-onset ALS, n (%)	35 (77.8)
Bulbar-onset ALS, n (%)	10 (22.2)
ALSFRS-R score (mean ± SD)	29.22 ± 4.92
Norris scale score (mean ± SD)	85.56 ± 15.55
ALSAQ-40 total score (mean ± SD)	36.31 ± 16.15
Arterial hypertension, n (%)	19 (42.2)
Other comorbidities, n (%)	21 (46.7)

ALS—amyotrophic lateral sclerosis; ALSFRS-R—revised ALS Functional Rating Scale; ALSAQ-40—ALS Assessment Questionnaire, 40-item version (higher scores denote worse quality of life); SD—standard deviation.

**Table 2:** Characteristics of the autologous cell product in patients who received all three administrations (*n* = 42).

Parameter	Range	Median	Mean ± SD
Bone marrow aspirate volume, mL	44–156	—	122.0 ± 22.7
MNC yield, 1st procedure (×10^6^)	38–560	180.5	207.9 ± 112.9
MNC yield, 2nd procedure (×10^6^)	68–504	170.3	187.8 ± 93.9
MNC yield, 3rd procedure (×10^6^)	18.5–538	178.0	205.7 ± 121.9
Cumulative MNC yield (×10^6^)	294–1320	590.7	601.5 ± 235.0
LIN^−^ cells administered, 1st procedure (×10^6^)	1.0–30.0	4.45	6.47 ± 6.05
LIN^−^ cells administered, 2nd procedure (×10^6^)	0.5–25.0	4.25	6.99 ± 6.31
LIN^−^ cells administered, 3rd procedure (×10^6^)	0.3–42.7	5.20	7.06 ± 7.22
Cumulative LIN^−^ cell dose (×10^6^)	2.8–65.0	17.7	20.5 ± 14.4
LIN^−^/MNC ratio, %	0.5–6.9	3.3	3.3 ± 1.6

MNC—mononuclear cell; LIN^−^—lineage-negative cells. MNC counts refer to cells isolated from the bone marrow aspirate; MNCs were not administered to patients. Values are given as ranges, medians and means ± standard deviation (SD); the LIN^−^/MNC ratio was calculated per patient from cumulative values. For the bone marrow aspirate volume, only the range and mean ± SD were recorded in the procedural documentation.

**Table 3:** Baseline characteristics of the longitudinal efficacy cohort stratified by total MNC yield.

Variable	All (*n* = 23)	Stratum A (<600 × 10^6^, *n* = 13)	Stratum B (≥600 × 10^6^, *n* = 10)	*p*
Age, years (mean ± SD)	53.61 ± 10.81	53.85 ± 11.57	53.30 ± 10.35	0.641
Female sex, n (%)	11 (47.8)	10 (76.9)	1 (10.0)	**0.003**
Male sex, n (%)	12 (52.2)	3 (23.1)	9 (90.0)	**0.003**
Disease duration, months (mean ± SD)	31.35 ± 29.07	33.92 ± 33.59	28.00 ± 23.23	0.684
Limb-onset ALS, n (%)	18 (78.3)	9 (69.2)	9 (90.0)	0.339
Bulbar-onset ALS, n (%)	5 (21.7)	4 (30.8)	1 (10.0)	0.339
ALSFRS-R score (mean ± SD)	30.78 ± 5.30	29.38 ± 5.28	32.60 ± 4.99	0.170
Norris scale score (mean ± SD)	90.65 ± 16.43	89.69 ± 17.96	91.90 ± 15.06	0.926
ALSAQ-40 total score (mean ± SD)	34.35 ± 14.61	38.75 ± 14.83	29.06 ± 13.12	0.113
Cumulative MNC yield (×10^6^)	594.5 ± 255.0	425.3 ± 106.9	814.3 ± 220.7	**<0.001**
Cumulative LIN^−^ dose (×10^6^)	22.44 ± 14.92	15.38 ± 9.94	31.61 ± 15.73	**0.006**
Arterial hypertension, n (%)	8 (34.8)	4 (30.8)	4 (40.0)	0.685
Other comorbidities, n (%)	10 (43.5)	7 (53.8)	3 (30.0)	0.402

Statistically significant values are shown in bold. Continuous variables were compared with the Mann–Whitney U test and categorical variables with Fisher’s exact test.

**Table 4:** Baseline characteristics of the longitudinal efficacy cohort stratified by cumulative administered LIN^−^ cell dose.

Variable	All (*n* = 23)	Stratum I (<15 × 10^6^, *n* = 9)	Stratum II (≥15 × 10^6^, *n* = 14)	*p*
Age, years (mean ± SD)	53.61 ± 10.81	55.22 ± 9.58	52.57 ± 11.77	0.548
Female sex, n (%)	11 (47.8)	6 (66.7)	5 (35.7)	0.214
Male sex, n (%)	12 (52.2)	3 (33.3)	9 (64.3)	0.214
Disease duration, months (mean ± SD)	31.35 ± 29.07	30.89 ± 35.19	31.64 ± 25.85	0.657
Limb-onset ALS, n (%)	18 (78.3)	6 (66.7)	12 (85.7)	0.343
Bulbar-onset ALS, n (%)	5 (21.7)	3 (33.3)	2 (14.3)	0.343
ALSFRS-R score (mean ± SD)	30.78 ± 5.30	29.67 ± 4.80	31.50 ± 5.65	0.431
Norris scale score (mean ± SD)	90.65 ± 16.43	94.22 ± 13.26	88.36 ± 18.28	0.416
ALSAQ-40 total score (mean ± SD)	34.35 ± 14.61	38.89 ± 12.99	31.20 ± 15.32	0.170
Cumulative MNC yield (×10^6^)	594.5 ± 255.0	399.1 ± 119.6	720.0 ± 240.5	**<0.001**
Cumulative LIN^−^ dose (×10^6^)	22.44 ± 14.92	8.78 ± 3.52	31.22 ± 12.53	**<0.001**
Arterial hypertension, n (%)	8 (34.8)	3 (33.3)	5 (35.7)	1.000
Other comorbidities, n (%)	10 (43.5)	4 (44.4)	6 (42.9)	1.000

Statistically significant values are shown in bold. Continuous variables were compared with the Mann–Whitney U test and categorical variables with Fisher’s exact test.

**Table 5:** Baseline characteristics of all treated patients, completers and non-completers of the 24-week assessment schedule.

Variable	All Treated (*n* = 42)	Completers (*n* = 23)	Non-Completers (*n* = 19)	*p*
Age, years	54.10 ± 8.94	53.61 ± 10.81	54.68 ± 6.20	0.690
Disease duration, months	28.64 ± 25.55	31.35 ± 29.07	25.37 ± 20.82	0.443
ALSFRS-R score	29.52 ± 4.99	30.78 ± 5.30	28.00 ± 4.24	0.067
Norris scale score	86.88 ± 14.82	90.65 ± 16.43	82.32 ± 11.41	0.061
Cumulative MNC yield (×10^6^)	601.5 ± 235.0	594.5 ± 255.0	610.0 ± 214.9	0.832
Cumulative LIN^−^ dose (×10^6^)	20.52 ± 14.44	22.44 ± 14.92	18.20 ± 13.88	0.347

Values are means ± SD. Non-completers were not identified at patient level in the baseline database; their values were derived from the summary statistics of all treated patients and of completers (post hoc). p—completers versus non-completers (Welch’s *t*-test). MNC—mononuclear cell; LIN^−^—lineage-negative cells; ALSFRS-R—revised ALS Functional Rating Scale.

**Table 6:** Number of patients with available assessments and mean Norris and ALSFRS-R scores at each time point (all treated patients).

Week	Norris, *n*	Norris, Mean	ALSFRS-R, *n*	ALSFRS-R, Mean
0	42	86.88	42	29.52
4	39	87.31	38	29.37
6	40	86.02	40	28.65
7	40	86.02	40	28.68
10	40	85.52	40	28.15
12	40	84.00	40	27.32
13	40	84.00	40	27.32
16	34	83.94	34	27.91
24 (completers only)	23	—	23	28.27

Administrations took place at weeks 0, 6 and 12. Week 24 data are available only for completers (Norris median 82 points; see Section 3.2). Means are unadjusted and based on all available observations at each time point.

**Table 7:** Safety and follow-up summary for the 42 patients who received three administrations.

Item	Finding
Bone marrow aspirations	126 (42 patients × 3)
Intrathecal administrations of LIN^−^ cells	126 (42 patients × 3)
CSF samples obtained (before and 7 days after each administration)	245 of 252 planned
Post-dural puncture headache, back pain, fever, infection	Not systematically recorded
Prespecified immediate or delayed procedure-related events	None recorded
Serious adverse events	None recorded
Deaths within 90 weeks of the first administration	9 of 42; none before week 24; causes not adjudicated; relationship to study procedures not formally assessed
Patients with scale assessments at week 24	23 of 42
Vital status available to week 90	42 of 42; telephone contact with patients or caregivers, not on a standardised schedule
Structured enquiry about adverse events after discontinuation	Not performed

LIN^−^—lineage-negative; CSF—cerebrospinal fluid. ‘None recorded’ denotes the absence of any documented event and not a formally adjudicated absence of harm.

## Data Availability

The data presented in this study are available on reasonable request from the corresponding author. The data are not publicly available because of restrictions related to patient privacy.

## Supplementary Materials

The following supporting information can be downloaded at https://www.mdpi.com/article/10.3390/jcm15207937/s1. Figure S1: Kaplan–Meier survival estimates according to the number of LIN^−^ cells administered at the first procedure (exploratory post hoc analysis). Group I (blue) received <5 × 10^6^ cells (*n* = 23). Group II (red) received ≥5 × 10^6^ cells (*n* = 19), of whom three were censored within the first 10 weeks, leaving 16 at risk when the single death in this subgroup occurred at week 75. Gehan–Wilcoxon *p* = 0.033; log-rank *p* = 0.035. Circles denote deaths and tick marks censored observations; observations were censored at week 90. This was one of approximately 100 uncorrected comparisons, one arm contributes a single event, and the result does not meet the corrected significance threshold defined in Section 2.7. It is presented for completeness and is hypothesis-generating only. Table S1: Completed TREND (Transparent Reporting of Evaluations with Nonrandomized Designs) checklist, with the location of each item in the manuscript. Table S2: CSF BDNF concentrations before and 7 days after each administration (all patients with paired samples). Table S3: Associations between the number of administered LIN^−^ cells and CSF BDNF.
