# Supplementary material for: Functional Status, Quality of Life and Survival After Repeated Intrathecal Administration of Autologous Lineage-Negative Cells in Amyotrophic Lateral Sclerosis
Source: J Clin Med. 2026 Oct 14;15(20):7937. doi: 10.3390/jcm15207937 (PMC13705995; doi:10.3390/jcm15207937)
Supplement: Supplementary file 1 [file jcm-15-07937-s001.zip › jcm-4568927-supplementary.pdf]

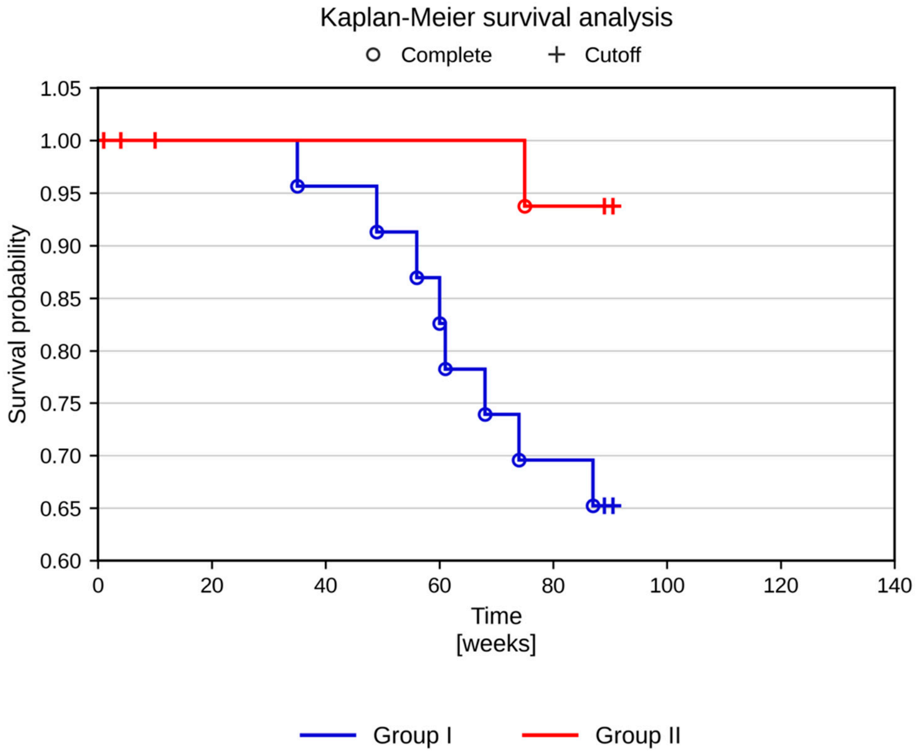

Figure S1. Kaplan–Meier survival estimates according to the number of LIN− cells administered at the first procedure (exploratory post hoc analysis). Group I (blue) received <5 × 10^6 cells (n = 23). Group II (red) received ≥5 × 10^6 cells (n = 19), of whom three were censored within the first 10 weeks, leaving 16 at risk when the single death in this subgroup occurred at week 75. Gehan–Wilcoxon p = 0.033; log-rank p = 0.035. Circles denote deaths and tick marks censored observations; observations were censored at week 90. This was one of approximately 100 uncorrected comparisons, one arm contributes a single event, and the result does not meet the corrected significance threshold defined in Section 2.7. It is presented for completeness and is hypothesis-generating only.

Table S1. Completed TREND (Transparent Reporting of Evaluations with Nonrandomized Designs) checklist, with the location of each item in the manuscript.

Pg # gives the page number(s) of the manuscript followed, in brackets, by the section, table or figure. "✓" – reported; "Partly" – partly reported; "N/A" – not applicable to a single-arm, non-randomised design of a biological intervention. Bracketed codes in the "Reported?" column refer to the explanatory notes below the checklist.

Paper Section/ Topic	Item No	Descriptor	Reported? ✓	Pg #
**Title and Abstract**				
Title and Abstract	1	• Information on how unit were allocated to interventions	✓	1 (Abstr.)
		• Structured abstract recommended	✓ [1b]	1–2 (Abstr.)
		• Information on target population or study sample	✓	1 (Abstr.)
**Introduction**				
Background	2	• Scientific background and explanation of rationale	✓	3–4 (1)
		• Theories used in designing behavioral interventions	N/A [2b]	3 (1)

**Methods**				
Participants	3	• Eligibility criteria for participants, including criteria at different levels in recruitment/sampling plan (e.g., cities, clinics, subjects)	✓	5 (2.2)
		• Method of recruitment (e.g., referral, self-selection), including the sampling method if a systematic sampling plan was implemented	Partly [3b]	5 (2.2)
		• Recruitment setting	✓ [3c]	4–5 (2.1)
		• Settings and locations where the data were collected	✓	5, 7, 9 (2.1, 2.3, 2.6)
Interventions	4	• Details of the interventions intended for each study condition and how and when they were actually administered, specifically including:	✓	5, 7–8 (2.1, 2.4)
		○ Content: what was given?	✓	7–8 (2.4)
		○ Delivery method: how was the content given?	✓	8 (2.4)
		○ Unit of delivery: how were the subjects grouped during delivery?	✓ [4c]	5, 8 (2.1, 2.4)
		○ Deliverer: who delivered the intervention?	Partly [4d]	7–8 (2.4)
		○ Setting: where was the intervention delivered?	✓	5, 8 (2.1, 2.4)
		○ Exposure quantity and duration: how many sessions or episodes or events were intended to be delivered? How long were they intended to last?	✓	5, 7 (2.1, 2.3)
		○ Time span: how long was it intended to take to deliver the intervention to each unit?	✓	5, 7 (2.1, 2.3)
		○ Activities to increase compliance or adherence (e.g., incentives)	N/A [4h]	—
Objectives	5	• Specific objectives and hypotheses	✓	4 (1)
Outcomes	6	• Clearly defined primary and secondary outcome measures	Partly [6a]	5, 7, 9 (2.1, 2.3, 2.6)
		• Methods used to collect data and any methods used to enhance the quality of measurements	✓	7, 9 (2.3, 2.5, 2.6)
		• Information on validated instruments such as psychometric and biometric properties	✓	7 (2.3)
Sample Size	7	• How sample size was determined and, when applicable, explanation of any interim analyses and stopping rules	✓ [7]	10 (2.7)
Assignment Method	8	• Unit of assignment (the unit being assigned to study condition, e.g., individual, group, community)	✓ [8a]	4–5 (2.1)
		• Method used to assign units to study conditions, including details of any restriction (e.g., blocking, stratification, minimization)	N/A [8b]	5 (2.1, 2.2)
		• Inclusion of aspects employed to help minimize potential bias induced due to non-randomization (e.g., matching)	N/A [8c]	10–11, 26 (2.7, 3.8)
Blinding (masking)	9	• Whether or not participants, those administering the interventions, and those assessing the outcomes were blinded to study condition assignment; if so, statement regarding how the blinding was accomplished and how it was assessed.	✓ [9]	4, 33 (2.1, 4.8)
Unit of Analysis	10	• Description of the smallest unit that is being analyzed to assess intervention effects (e.g., individual, group, or community)	✓ [10a]	5 (2.1)
		• If the unit of analysis differs from the unit of assignment, the analytical method used to account for this (e.g., adjusting the standard error estimates by the design effect or using multilevel analysis)	N/A [10b]	—

Statistical Methods	11	• Statistical methods used to compare study groups for primary methods outcome(s), including complex methods of correlated data	✓ [11a]	9–10 (2.7)
		• Statistical methods used for additional analyses, such as a subgroup analyses and adjusted analysis	✓	10–11 (2.7)
		• Methods for imputing missing data, if used	✓ [11c]	11 (2.7)
		• Statistical software or programs used	✓	9 (2.7)
**Results**				
Participant flow	12	• Flow of participants through each stage of the study: enrollment, assignment, allocation, and intervention exposure, follow-up, analysis (a diagram is strongly recommended)	✓	5–6, 11–12 (Fig. 1, 3.1)
		○ Enrollment: the numbers of participants screened for eligibility, found to be eligible or not eligible, declined to be enrolled, and enrolled in the study	Partly [12a]	5–6, 11 (Fig. 1, 3.1)
		○ Assignment: the numbers of participants assigned to a study condition	✓ [12b]	5–6, 11 (Fig. 1, 3.1)
		○ Allocation and intervention exposure: the number of participants assigned to each study condition and the number of participants who received each intervention	✓	6, 11, 13 (Fig. 1, 3.1, Table 2)
		○ Follow-up: the number of participants who completed the follow-up or did not complete the follow-up (i.e., lost to follow-up), by study condition	✓	6, 11–12, 15 (Fig. 1, 3.1, Table 6)
		○ Analysis: the number of participants included in or excluded from the main analysis, by study condition	✓	5–6, 12 (2.1, Fig. 1, 3.1)
		• Description of protocol deviations from study as planned, along with reasons	Partly [12f]	11–12 (3.1)
Recruitment	13	• Dates defining the periods of recruitment and follow-up	✓	4 (2.1)
Baseline Data	14	• Baseline demographic and clinical characteristics of participants in each study condition	✓ [14a]	11–12 (3.1, Table 1)
		• Baseline characteristics for each study condition relevant to specific disease prevention research	✓	11–14 (Tables 1, 3, 4)
		• Baseline comparisons of those lost to follow-up and those retained, overall and by study condition	✓	12, 14 (3.1, Table 5)
		• Comparison between study population at baseline and target population of interest	Partly [14d]	32–34 (4.7, 4.8)
Baseline equivalence	15	• Data on study group equivalence at baseline and statistical methods used to control for baseline differences	N/A [15]	13–14, 33–34 (Tables 3, 4; 4.8)

Numbers analyzed	16	• Number of participants (denominator) included in each analysis for each study condition, particularly when the denominators change for different outcomes; statement of the results in absolute numbers when feasible	✓	10, 15 (2.7, Table 6, Figs 2–5)
		• Indication of whether the analysis strategy was "intention to treat" or, if not, description of how non-compliers were treated in the analyses	✓ [16b]	12, 26 (3.1, 3.8)
Outcomes and estimation	17	• For each primary and secondary outcome, a summary of results for each estimation study condition, and the estimated effect size and a confidence interval to indicate the precision	✓ [17a]	15–26 (3.2–3.8)
		• Inclusion of null and negative findings	✓	19–26 (3.3–3.8)
		• Inclusion of results from testing pre-specified causal pathways through which the intervention was intended to operate, if any	Partly [17c]	22–25 (3.6)
Ancillary analyses	18	• Summary of other analyses performed, including subgroup or restricted analyses, indicating which are pre-specified or exploratory	✓	21, 26, 35 (3.5, 3.8, Suppl.)
Adverse events	19	• Summary of all important adverse events or unintended effects in each study condition (including summary measures, effect size estimates, and confidence intervals)	✓	25–26 (3.7, Table 7)
**DISCUSSION**				
Interpretation	20	• Interpretation of the results, taking into account study hypotheses, sources of potential bias, imprecision of measures, multiplicative analyses, and other limitations or weaknesses of the study	✓	27–30 (4.1–4.4)
		• Discussion of results taking into account the mechanism by which the intervention was intended to work (causal pathways) or alternative mechanisms or explanations	✓	30–33 (4.5, 4.7)
		• Discussion of the success of and barriers to implementing the intervention, fidelity of implementation	✓	27, 33–34 (4.2, 4.8)
		• Discussion of research, programmatic, or policy implications	✓	34–35 (4.9, 5)
Generalizability	21	• Generalizability (external validity) of the trial findings, taking into account the study population, the characteristics of the intervention, length of follow-up, incentives, compliance rates, specific sites/settings involved in the study, and other contextual issues	✓	33–34 (4.8)
Overall Evidence	22	• General interpretation of the results in the context of current evidence and current theory	✓	31–32, 35 (4.6, 5)

Explanatory notes

[1b] The Journal of Clinical Medicine requires a single-paragraph abstract (maximum 200 words); the abstract nevertheless follows the structure background – methods – results – conclusions and states the design (open-label, single-arm), the study sample, the outcomes and the harms.

[2b] Not applicable: the intervention is biological (autologous cell therapy), not behavioural. The biological rationale (paracrine neurotrophic and immunomodulatory action of bone marrow-derived LIN− cells) is given in the Introduction.

[3b] Patients with sporadic ALS were qualified by the study board after at least three months of observation and riluzole treatment; no systematic sampling plan was used.

[3c] Single centre (Pomeranian Medical University in Szczecin); all procedures and assessments were carried out during hospitalisation and scheduled follow-up visits at the same centre.

[4c] The intervention was delivered to individual patients; there was no grouping of participants.

[4d] All procedures (bone marrow harvest, GMP isolation of LIN− cells and intrathecal administration) were performed by the study team at the single participating centre, as described previously (refs. 31, 33); the professional roles of individual operators are not specified in the manuscript.

[4h] Not applicable: no incentives or specific activities to increase adherence were used; delivery of the intervention is documented in Section 3.1 and Table 2 (126 administrations in 42 patients).

[6a] All outcomes (Norris scale, ALSFRS-R, ALSAQ-40, survival, CSF neurotrophins, safety) are defined, but, given the exploratory design, no formal primary/secondary hierarchy was designated; this is reflected in the exploratory interpretation of all results.

[7] Section 2.7: the sample size was not based on a formal power calculation because the study was exploratory; no interim analyses or stopping rules were prespecified.

[8a] Unit of assignment: the individual patient. All qualified patients were assigned to the same intervention (single-arm design).

[8b] Not applicable: single-arm study without a comparison group; there was no allocation procedure, blocking, stratification or minimisation. Patients were not stratified a priori by cell yield or dose (Section 2.2).

[8c] Not applicable in the strict sense (no comparison group, no matching). Potential bias arising from the uncontrolled design and from attrition is addressed by sensitivity analyses in all 42 treated patients (linear mixed models, comparison with individual pre-treatment progression rate ΔFS) and discussed in Section 4.8.

[9] No blinding: open-label design; participants, those administering the intervention and outcome assessors were aware of treatment. The consequences are discussed in Sections 4.3 and 4.8.

[10a] The unit of analysis is the individual patient, identical to the unit of assignment.

[10b] Not applicable: the unit of analysis does not differ from the unit of assignment.

[11a] No between-group comparison of study conditions (single arm); within-patient longitudinal change was analysed with Friedman ANOVA and Wilcoxon tests, and post hoc strata were compared with Mann–Whitney U / Kruskal–Wallis tests.

[11c] No imputation was performed. Primary longitudinal analyses used complete cases; sensitivity analyses used linear mixed models with all available observations under a missing-at-random assumption.

[12a] 45 patients were qualified, 42 treated; the number of patients screened before qualification is not available in the analysed database.

[12b] Single study condition: all 42 treated patients received the same intervention (three intrathecal administrations).

[12f] Three qualified patients did not receive cells; 19 treated patients discontinued scheduled assessments because of functional deterioration. Individual reasons and dates of discontinuation were not documented in the analysed database; this is stated in Section 3.1.

[14a] Baseline characteristics are reported for the single study cohort (Table 1) and for the longitudinal efficacy cohort (Section 3.1).

[14d] No formal comparison with the target population was performed; the observed rate of ALSFRS-R decline is discussed in relation to untreated ALS populations (PRO-ACT database, ref. 48), and the

representativeness of the cohort (completers with milder disease, clinically defined sporadic ALS) is discussed in Section 4.8.

[15] Not applicable: single-arm study. Baseline differences between the post hoc strata (MNC yield, LIN− dose) are reported in Tables 3 and 4 and discussed as potential confounders in Section 4.8; no statistical adjustment was feasible.

[16b] No intention-to-treat analysis was possible because the three qualified but untreated patients contributed no data; the main analyses are per-protocol (completers), with sensitivity analyses in all 42 treated patients (Section 3.8).

[17a] Results are given for the single study condition; effect estimates with 95% confidence intervals (mixed-model slopes) are given in Section 3.8.

[17c] No causal pathway was prespecified; the relationship between administered cell numbers and CSF neurotrophins (a hypothesised mechanism) was examined in exploratory analyses (Section 3.6, Tables S2–S3).

From: Des Jarlais, D. C., Lyles, C., Crepaz, N., & the Trend Group (2004). Improving the reporting quality of nonrandomized evaluations of behavioral and public health interventions: The TREND statement. *American Journal of Public Health*, 94, 361-366. For more information, visit: http://www.cdc.gov/trendstatement/

Table S2. Cerebrospinal fluid BDNF concentrations (pg/mL) before (day 0) and 7 days after each intrathecal administration of LIN^{-} cells, all patients with paired samples.

Cycle	n (paired)	Day 0: median (IQR)	Day 0: mean ± SD	Day 7: median (IQR)	Day 7: mean ± SD	Change day 7 − day 0: median (IQR)	Wilcoxon p
I	42	3.75 (3.01–4.70)	21.2 ± 93.1	3.90 (3.16–5.27)	44.4 ± 156.7	0.07 (−0.58 to 0.82)	0.51
II	41	4.23 (1.26–7.26)	5.4 ± 4.9	4.89 (2.12–7.78)	10.9 ± 25.5	0.27 (−2.72 to 4.08)	0.65
III	39	7.57 (4.72–12.64)	12.3 ± 19.2	8.61 (5.66–14.04)	17.2 ± 30.5	0.82 (−1.23 to 4.16)	0.145

IQR — interquartile range; SD — standard deviation. Post hoc analysis of all available paired samples; no correction for multiplicity. Compared with day 0 of cycle I (Wilcoxon signed-rank test): cycle II day 0, median difference −0.57 pg/mL, p = 0.44; cycle II day 7, 1.01, p = 0.15; cycle III day 0, 1.98, p = 0.0009; cycle III day 7, 4.00, p = 0.0008.

Table S3. Associations between the cumulative number of LIN^{-} cells administered at the third procedure and CSF BDNF in cycle III (post hoc).

Analysis	n	Estimate	p
Spearman ϱ, cell number vs day-7 BDNF	39	0.14	0.39
Spearman ϱ, cell number vs day-0 BDNF	39	−0.14	0.38
Spearman ϱ, cell number vs change (day 7 – day 0)	39	0.40	0.012

Partial Spearman ϱ, cell number vs day-7 BDNF adjusted for day-0 BDNF	39	0.28	0.089
Spearman ϱ, cell number vs day-7 BDNF after exclusion of the three highest BDNF values	36	−0.05	0.79
Log–log regression: coefficient for cell number (adjusted for day-0 BDNF)	39	0.27 (95% CI −0.01 to 0.55)	0.058
Log–log regression: coefficient for day-0 BDNF	39	0.32 (95% CI 0.05 to 0.59)	0.022

The correlation reported in the main text (r = 0.75, n = 22) was calculated in a subset of patients and was not reproduced in the full sample.
